# Modular Architecture and Unique Teichoic Acid Recognition Features of Choline-Binding Protein L (CbpL) Contributing to Pneumococcal Pathogenesis

**DOI:** 10.1038/srep38094

**Published:** 2016-12-05

**Authors:** Javier Gutiérrez-Fernández, Malek Saleh, Martín Alcorlo, Alejandro Gómez-Mejía, David Pantoja-Uceda, Miguel A. Treviño, Franziska Voß, Mohammed R. Abdullah, Sergio Galán-Bartual, Jolien Seinen, Pedro A. Sánchez-Murcia, Federico Gago, Marta Bruix, Sven Hammerschmidt, Juan A. Hermoso

**Affiliations:** 1 Department of Crystallography and Structural Biology, “Rocasolano” Institute of Physical-Chemistry, CSIC, Serrano 119, E-28006-Madrid, Spain; 2 Department Genetics of Microorganisms, Interfaculty Institute for Genetics and Functional Genomics, Ernst Moritz Arndt University of Greifswald, D-17487 Greifswald, Germany; 3 Department of Biological Physical Chemistry. “Rocasolano” Institute of Physical-Chemistry, CSIC, Serrano 119, E-28006-Madrid, Spain; 4 Department of Biomedical Sciences, Unidad Asociada al IQM-CSIC, Universidad de Alcalá, E-28871 Alcalá de Henares, Madrid, Spain

## Abstract

The human pathogen *Streptococcus pneumoniae* is decorated with a special class of surface-proteins known as choline-binding proteins (CBPs) attached to phosphorylcholine (PCho) moieties from cell-wall teichoic acids. By a combination of X-ray crystallography, NMR, molecular dynamics techniques and *in vivo* virulence and phagocytosis studies, we provide structural information of choline-binding protein L (CbpL) and demonstrate its impact on pneumococcal pathogenesis and immune evasion. CbpL is a very elongated three-module protein composed of (i) an Excalibur Ca^2+^-binding domain -reported in this work for the very first time-, (ii) an unprecedented anchorage module showing alternate disposition of canonical and non-canonical choline-binding sites that allows vine-like binding of fully-PCho-substituted teichoic acids (with two choline moieties per unit), and (iii) a Ltp_Lipoprotein domain. Our structural and infection assays indicate an important role of the whole multimodular protein allowing both to locate CbpL at specific places on the cell wall and to interact with host components in order to facilitate pneumococcal lung infection and transmigration from nasopharynx to the lungs and blood. CbpL implication in both resistance against killing by phagocytes and pneumococcal pathogenesis further postulate this surface-protein as relevant among the pathogenic arsenal of the pneumococcus.

The Gram-positive bacterium *Streptococcus pneumoniae* is one of the most important human pathogens and a widely distributed commensal of the upper respiratory tract. However, under appropriate conditions, pneumococcal colonization can progress to invasive disease such as sepsis and meningitis^1^, and thus become lethal. For this reason, antibiotics and vaccines are designed to limit the dramatic effects of the bacteria in such cases. Many pneumococcal surface proteins are virulence factors contributing to pathogenesis and playing a role in colonization and disease. Thus, these proteins represent challenging candidates for the development of new therapeutic targets against the bacterium^2^,^3^.

Four families of surface proteins are present on the cell surface of *S. pneumoniae*, which are related to three different attachment modes to the cell wall, composed of peptidoglycan, wall teichoic acids (WTA) and lipoteichoic acids (LTA). The latter two are decorated with phosphorylcholine (PCho) residues. Besides lipoproteins, sortase-anchored LPxTG proteins and surface-exposed moonlighting proteins that exist in other Gram-positive bacteria, the pneumococcus displays Choline-Binding Proteins (CBPs)^4^.

The number of CBPs varies from 13 to 16 depending on the pneumococcal strain and a large body of literature about this family indicates CBPs play key roles in cell wall physiology, in the colonization process and in the host cells interaction commanding to the disease transition: LytA is a virulence factor involved in autolysis as well as in fratricidal- and penicillin-induced lysis^5^ and also participating in capsule removal to combat antimicrobial peptides^6^, Pce (also named CbpE) selectively modifies PCho distribution on bacterial surface to block recognition by immune system and also hydrolyses the platelet-activating factor (PAF) to suppress inflammation during airway infection^7^, PspC is the major adhesin of *S. pneumoniae*^8^ able to bind Factor H^9^, vitronectin^10^, thrombospondin-1^11^ and secretory IgA or the ectodomain of the polymeric immunoglobulin receptor (pIgR) during invasion in nasopharynx^12^,^13^, CbpD and LytC are murolytic CBPs that are key components in the virulence mechanism named fratricide^14^,^15^ and CbpF, a non-enzymatic CBP, inhibits activity of autolysin LytC^16^. A common feature of the CBP is that they share a modular organization that includes, at least, the choline-binding module (CBM) and a module exerting a biological function. The CBM interacts with choline molecules from WTA and LTA in a non-covalent manner, attaching the whole protein to the peptidoglycan layer. In general, the CBM is present in the C-terminal part of the protein with the exception of LytB and LytC, where the CBM is located at the N-terminal part^15^. CbpL is the only one having the CBM sandwiched between two functional modules located at both extremes. In CBPs the CBM is composed of three to eighteen repeated sequences of approximately 20 amino acids forming the different choline-binding sites^17^. These sites are integrated by specific configurations of aromatic residues that stabilize the choline molecules from WTA and LTA through cation-*π* interactions^18^.

Here, we describe the functional characterization and the three-dimensional structure of CbpL from *S. pneumoniae*. CbpL is a 332 residues-long protein with a molecular weight of 37.6 kDa. A leader peptide comprising the first 26 amino acids is involved in the transport and orientation of the protein at its final location (Fig. 1 and Fig. S1). The functional protein has 3 well-differentiated domains connected by linker regions that provide flexibility to the general structure. The N-terminal domain, ranging from Glu27 to Glu67, includes an Excalibur domain (from *ex*tracellular *cal*cium-*b*inding *r*egion, pfam05901). Although its function is unknown, in streptococci and staphylococci the Excalibur domain is found associated to various low-complexity domains, which might be involved in adhesion and colonization processes^19^. While immunogenic properties of these proteins have not been investigated so far, they might represent interesting vaccine candidates^19^. Some features of the Excalibur domains include (i) two absolutely conserved Cys residues and (ii) the conserved DxDxDGxxCE motif, which is strikingly similar to the Ca^2+^-binding region of calmodulin. Following a 27-residue long linker, CbpL has a central region (from Gly95-Glu270) displaying the CBM. Finally, after another linker region of 14 residues, there is a Ltp_Lipoprotein domain (pfam07553) at the C-terminal end, ranging from Asn283 to Asp332. This domain is a characteristic property of lipoproteins from temperate phages^20^. The fact that such protein domain composition is a unique property of CbpL compared to other CBPs prompted us to disclose its structure and biological functionalities.

Using a combination of X-ray crystallography, NMR and molecular modeling tools, we provide a structural model accounting for all the different modules of the whole protein as well as for its anchoring interaction with WTA/LTA. This structural model is complemented by *in vivo* virulence and phagocytosis studies to provide a full approach on the pathophysiological relevance of CbpL for pneumococcal pathogenesis.

## Results

### CbpL is a choline-binding protein decorating the pneumococcal cell surface

**Figure 1:**
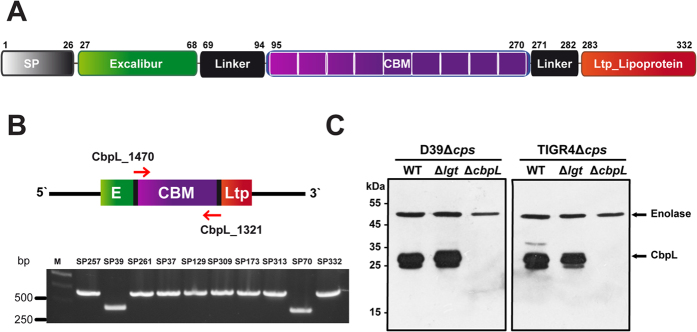
Expression of CbpL by pneumococci. (**A**) Modular organization of CbpL. (**B**) Molecular analysis of the variable numbers of choline-binding repeats in CbpL. A PCR using primers cbpL_1470 and cbpL_1321 was carried out using chromosomal DNA from various pneumococci as DNA template (Table S1) to amplify the *cbpL* gene. The length of PCR products was approximately 660 bp and 460 bp, respectively. Primer CbpL_1470 starts downstream of the Excalibur module and the reverse primer CbpL_1321 starts in the 3′end of the CBM module. (**C**) Immunoblot analysis of CbpL expression in wild-type, *lgt*- and *cbpL*-mutant pneumococci using specific polyclonal anti-CbpL antibodies. Enolase was used as a control.

The gene encoding CbpL (e.g. *SPD_0579* in D39 and *SP_0667* in TIGR4; protein accession YP_816078.1 and NP_345172.1) is located in a region encompassing genes encoding the zinc metalloprotease B (ZmpB) and *para*-aminobenzoic acid synthetase (PabB)^21^,^22^, in the upstream region (Fig. S2). Downstream of *cbpL* the glucokinase (Gki) and thymidylate synthase (ThyA) encoding genes are found. The *in silico* analysis suggested different genomic organizations when e.g. comparing D39 and TIGR4 (Fig. S2). In D39 a transcription terminator is predicted in the 3′- end of *pabB* followed by promoter sequence (AGACTCTTGAATTGCTGAAATAAGTTTGATAAAATAATATTGAAATCGAT) upstream *cbpL*. Downstream *cbpL* a transcription terminator (TGAGAGAGGAAAATGCTAACCTAGAGTTAGTAATGTTCCTCTTTTA) is found (Fig. S2). In contrast, the *cbpL* locus in TIGR4 is suggested to be polycistronic (Fig. S2) with a promoter sequence upstream of *pabB* and downstream of *cbpL*. In TIGR4 the gene *sp_0666*, encoding a putative pyrimidine utilization protein^21^ is additionally annotated between *pabB* and *cbpL*. Remarkably, loss of function of ZmpB was correlated with a delocalization of some CBPs like CbpA, CbpE, CbpF, CbpJ and LytA^23^. CbpL modular organization is shown in Fig. 1A. The leader peptide (amino acid residues 1–26) important for secretion of CbpL is followed by a so-called Excalibur domain (amino acid residues 27–68; pfam05901), which has been proposed to bind Ca^2+^ as has been shown e.g. for YokF from *Bacillus subtilis*. The pneumococcal Excalibur domain shares conserved motifs with YokF but also e.g. with SA1617 from *Staphylococcus aureus*^19^. The CBM (aa 95 to 270) in the central part of the protein is connected via linker regions to the Excalibur domain in the N-terminal part and a Ltp_Lipoprotein domain (amino acid residues 283 to 332; pfam07553) located in the C-terminal part of CbpL. Ltp is a lipoprotein of temperate phages present in *Streptococcus thermophilus* preventing injection of DNA of infecting phages into the cytoplasm, a mechanism also known as superinfection exclusion^24^. The Ltp lipoprotein is located outside of the cytoplasmic membrane, expressed in the lysogenic phase of temperate phages^20^. Sequence comparisons indicated the high conservation of CbpL among pneumococcal isolates (Fig. S3). However, the number of repeats in the CBM, which are essential for cell-wall attachment, varies as indicated also by a PCR-based molecular analysis (Fig. 1B).

**Figure 2:**
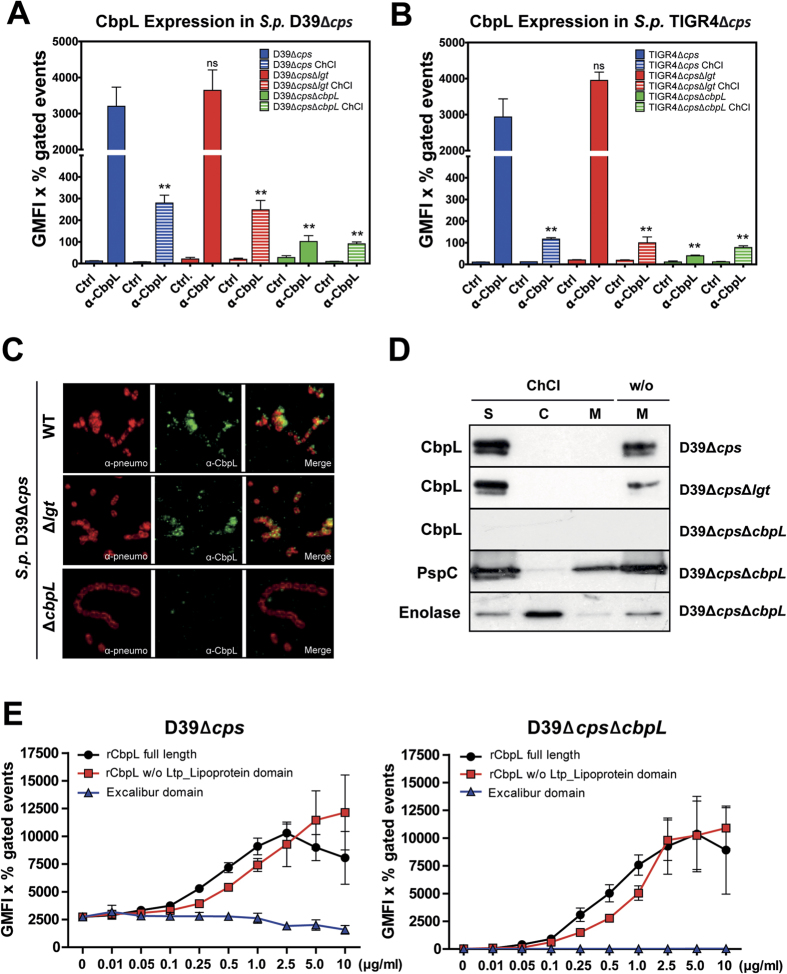
CbpL is a choline-binding protein exposed on the pneumococcal cell surface. (**A**,**B)** The surface-exposure of CbpL proven by flow cytometric analysis using polyclonal anti-CbpL antibodies as primary antibodies and secondary goat anti-mouse IgG coupled Alexa-Fluor-488 (Invitrogen). The surface abundance of CbpL was measured without and after ChCl treatment (10% ChCl for 30 min), which generally results in release of CBPs. The bar charts (**A**,**B**) demonstrate the increase in fluorescence intensity of the parental strains D39Δ*cps* or TIGR4Δ*cps* compared to the isogenic *cbpL*-mutants and controls (Ctrl). The *lgt*-mutants of D39Δ*cps* and TIGR4Δ*cps* showed similar fluorescence intensities compared to the parental strains D39Δ*cps* and TIGR4Δ*cps*, while ChCl treatment significantly reduced anti-CbpL binding, indicative of a of a loss of binding of CbpL on the surface. Graphs (**A**,**B**) show the results (GMFI multiplied with the number of positive events [%]) of three independent experiments ± S.D. *P < 0.05, and **P < 0.01 relative to the parental strains incubated with the 1^st^ and 2^nd^ antibody. Individual histograms and dot plots are depicted in Fig. S4. (**C**) Immunofluorescence microscopy of D39Δ*cps*, D39Δ*cps*Δ*lgt,* D39Δ*cps*Δ*cbpL* to show CbpL on the bacterial cell surface. Pneumococci were incubated with rabbit anti-pneumococcal IgG^70^ followed by secondary goat anti-rabbit IgG coupled Alexa-Fluor-568 (Invitrogen) to stain pneumococci (red fluorescence) and CbpL was detected with polyclonal anti-CbpL antibodies and secondary goat anti-mouse IgG coupled Alexa-Fluor-488. (**D**) Immunoblot analysis of pneumococcal protein fractions (S: supernatant; C: cytoplasmic; M: cell membrane) enriched prior or post-treatment of pneumococci with choline chloride (ChCl). Protein fractions were separated by SDS-PAGE and after blotting CbpL was detected using anti-CbpL polyclonal antibodies. Choline-binding protein PspC and enolase (cytoplasmic and moonlighting protein) were used as controls and detected with anti-PspC or anti-enolase antibodies^71^,^72^. **(E)** Dose-dependent reassociation of various recombinant CbpL peptides measured by flow cytometry post-incubation of CbpL-expressing D39Δ*cps* or CbpL-deficient D39Δ*cps*Δ*cbpL*. The graphs, showing the results (GMFI multiplied with the number of positive events [%]) of three independent experiments ± S.D, demonstrate reassociation of CbpL peptides to the pneumococcal cell surface when the CBM is present.

To confirm the surface-exposure of CbpL and to assess the impact of CbpL on pneumococcal pathogenesis, mutants were generated in the D39 and TIGR4 genetic background by allelic replacement (Fig. S2B). Immunoblot analysis using CbpL-specific antibodies generated in mice against recombinant CbpL (rCbpL) indicated the deficiency of CbpL in *cbpL*-mutants of non-encapsulated D39 and TIGR4 (Fig. 1C). All previously reported proteins of other bacterial species sharing a Ltp_Lipoprotein domain are lipoproteins containing the lipobox motif LxxC that are therefore translocated and membrane anchored by the action of the diacylglyceryl transferase Lgt. This enzyme, together with the lipoprotein specific signal peptidase Lsp, is essential to anchor lipoproteins properly in the membrane. The deficiency of the Lgt was shown to alter the molecular weight and localization of lipoproteins^25^. The Ltp_Lipoprotein in CbpL, however, is 48 aa in length (TIGR4) and lacks the recognition lipobox motif LxxC. In order to assess the potential binding of CbpL to the membrane, *lgt*-mutants were assessed for expression and surface exposure of CbpL. Immunoblots of *lgt*-mutants demonstrated no changes in the molecular weight of CbpL and it must be further noted that the protein amount was not substantially altered compared to isogenic wild-type pneumococci (Fig. 1C). To demonstrate the abundance of CbpL on the pneumococcal surface and to decipher any influence of surface localization via a modification by the Lgt, flow cytometry was exploited using anti-CbpL antibodies. The results indicated the absence of CbpL in the *cbpL*-mutants of D39Δ*cps*Δ*cbpL* and TIGR4Δ*cps*Δ*cbpL*. In addition, the abundance of CbpL was not altered in the *lgt*-mutants (Fig. 2A,B and Fig. S4). Immunofluorescence microscopy confirmed these data and indicated CbpL expression and surface-exposure for the wild-type and *lgt*-mutant but not for the *cbpL*-mutant (Fig. 2C). Interestingly, CbpL seems not to be evenly distributed over the surface as demonstrated in the immunofluorescence microscopy, however, the reason for this phenomenon is still unknown. To further indicate that the choline-binding protein (CBP) CbpL is non-covalently associated to the pneumococcal cell surface via the CBM, pneumococci were treated with choline chloride (ChCl) resulting in release of CBPs from the surface. The flow cytometric analysis demonstrated a significant reduction of CbpL in ChCl-treated bacteria (Fig. 2A,B and Fig. S4). In addition, the subcellular analysis of pneumococcal protein fractions by immunoblot analysis showed that CbpL is absent in the membrane fraction due to its release into the supernatant upon treatment with ChCl, whereas without ChCl treatment CbpL remained bound to pneumococci and can be detected in the membrane fraction (Fig. 2D). To test whether purified CbpL fragments containing the CBM are able to reassociate to the pneumococcal cell surface by interacting with the PCho, pneumococci were incubated with various recombinant CbpL domains and binding was analyzed by flow cytometry. The results revealed that rCbpL, consisting of the Excalibur domain, CBM and Ltp-Lipoprotein, and the Excalibur-CBM domain of CbpL reassociate to pneumococci, while the Excalibur domain did not (Fig. 2E). Taken together, these results indicate that CbpL is a CBP non-covently attached to the phosphorylcholine of pneumococcal teichoic acids.

### Crystal structure of the choline-binding module of CbpL in complex with choline

**Figure 3:**
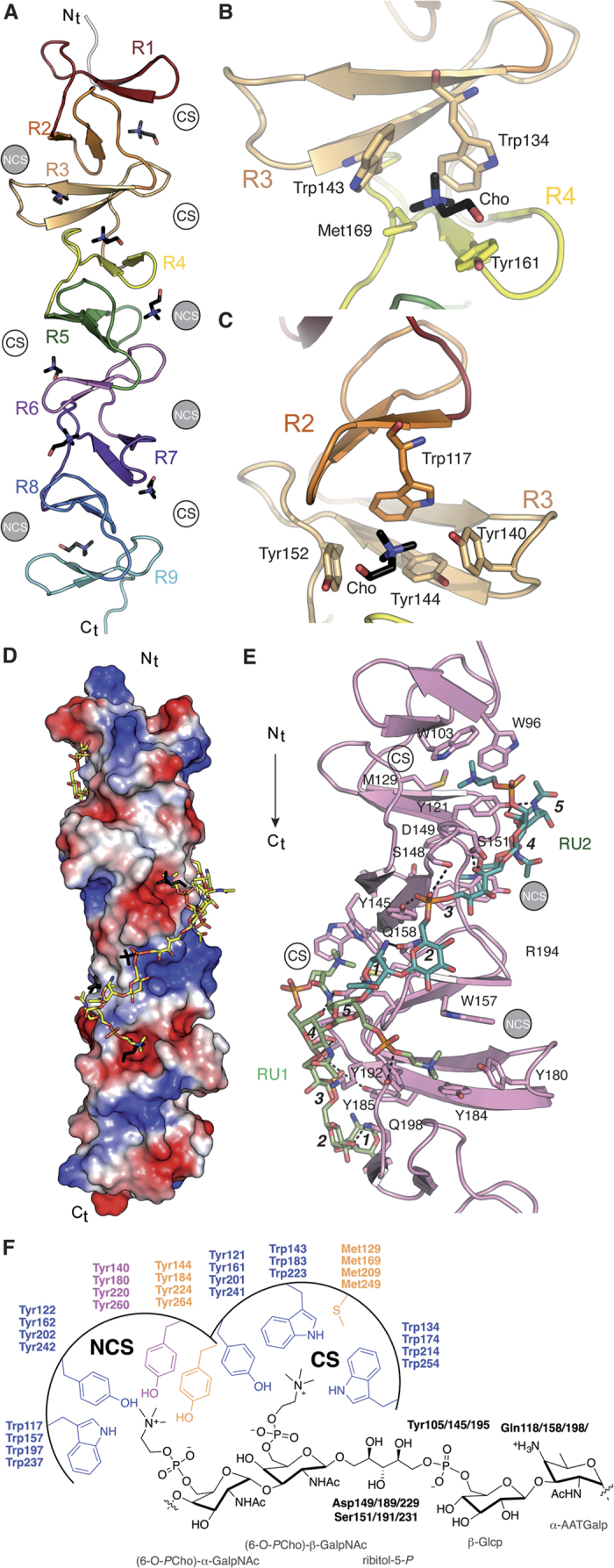
Crystal structure of the CBM and teichoic acid recognition of CbpL. (**A**) Ribbon representation of the overall fold of the CBM. Each choline-binding repeat is colored differently and labeled (R1–R9). Choline moieties attached to the choline-binding sites are represented as capped sticks with C atoms in black. Canonical (CS) and non-canonical (NCS) sites are labeled. (**B**) Molecular architecture of the canonical choline-binding site with critical residues represented as capped sticks and labeled. (**C**) Molecular architecture of the non-canonical choline-binding site with critical residues represented as capped sticks and labeled. (**D**) Electrostatic-potential surface of the crystal structure of CbpL_CBM_:choline complex with the docked model of three TA units (yellow sticks for C atoms) superimposed. Choline moieties and sulphate molecules from the CbpL_CBM_:choline complex are depicted as black sticks for comparison purposes. (**E**) Detailed view of the interactions between two docked TA repeating units (RU1 and RU2, light green sticks and dark green sticks respectively) and the CbpL_CBM_ (pink ribbon). Different sugars composing RU in TA are labeled (***1***: α-AATGalp, ***2***: β-Glcp, ***3***: ribitol-5-*P, **4***: (6-O-*P*Cho)-β-GalpNAc and ***5***: (6-O-*P*Cho)-α-GalpNAc). Residues involved in TA stabilization are depicted as capped sticks. Canonical sites (CS) and non-canonical sites (NCS) are labeled and polar interactions represented as dotted lines. (**F**) Scheme of the TA recognition by CS and NCS in CbpL. Residues involved in TA binding are conserved along the CbpL_CBM_ (labeled).

Crystallization trials with full-length CbpL systematically resulted in proteolysis of the sample through the linker regions and only the choline-binding module (CbpL_CBM_) was incorporated into the crystals (see Methods). The crystal structure of CbpL_CBM_:choline complex was solved at 1.7 Å resolution (Table 1 and Fig. S5) by the molecular replacement method, using the CBM of CbpF as initial model (PDB code 2V04). The crystal structure presents three residues of the N-term linker (Thr92-Ser94), the CBM that is composed of nine choline-binding repeats (residues Gly95-Glu270), and three more residues from the C-term linker (Val271-Ala273). The overall shape of the CbpL_CBM_ is approximately a triangular prism of 81 Å high and 22 Å side with the choline-binding sites placed along the three lateral faces of the prism (Fig. 3A). CbpL_CBM_ is folded in a left-handed superhelical arrangement of the choline-binding repeats, each of them comprising a β-hairpin followed by a loop and a coiled region that is linked to the next repeat, as previously reported in other CBPs^15^,^16^,^17^,^26^. The β-hairpin units stack along the structure (from N to C-terminal end) following a 120° counterclockwise rotation in such a way that choline-binding sites are formed at the interface of two consecutive β-hairpins. These sites are 26–28 Å separated from each other. According to DALI server^27^ the closest structural homologues of the CbpL_CBM_ are the CBM of pneumococcal autolysin LytC (PDB code 2WWD) and the CBM of CbpF (PDB code 2V05) (Fig. S6).

Structural and sequence analyses revealed that among the nine choline-binding repeats (R1-R9), none of them follows the consensus sequence GWXK-X_4–5_-WYY-φ-X_3–5_GXMX_2–3_, where X is any residue and φ is hydrophobic, as observed for other CBPs^16^,^17^. Instead, the repeats can be grouped in two main classes: the long repeats (21 to 23 residues-long; R1, R3, R5, R7 and R9) and the short repeats (17 residues-long; R2, R4, R6, R8) (Fig. S7). The long repeats are rich in aromatic residues (containing seven, except for the two extreme repeats R1 and R9 with five and four aromatic residues, respectively) while the short repeats have just three aromatic residues. This unique composition for the CbpL_CBM_ results in specific features in the choline-binding sites. The crystal structure of CbpL_CBM_:choline complex shows that eight choline-binding sites are available. Among them, four are canonical sites (CS), displaying the typical choline stabilization found in other CBPs, while the other four are non-canonical sites (NCS) (Fig. 3 and Fig. S8). In the CS the choline molecules are stabilized by cation-*π* interactions with three structurally conserved aromatic residues (two tryptophan residues from a β-hairpin and one tyrosine residue from the next β-hairpin) and by hydrophobic and electrostatic interactions with one methionine residue located at the bottom of the cavity (Fig. 3B). In the NCS the choline molecules are stabilized by cation-*π* interactions with four aromatic residues. In this case, only one tryptophan residue is provided by the first β-hairpin, two tyrosine residues come from the second β-hairpin (one of them from the turn of this β-hairpin, Tyr140 in Fig. 3C) and the fourth aromatic residue (Tyr152 in Fig. 3C) comes from the loop followed the β-hairpin and replaces the methionine residue found in CS (Fig. 3). Remarkably, CS and NCS alternate along the CbpL_CBM_ starting from a CS formed between R1 and R2 repeats (Fig. 3A). This is a unique property of CbpL that has not been observed in any other CBP reported to date.

### Differential teichoic-acid binding in CbpL

Docking calculations and molecular dynamics (MD) simulations with different WTA fragments and two whole repeating units (RU) suggested a plausible binding mode to both CS and NCS (see methods). In the computed model of CbpL_CBM_ in complex with two WTA units (Fig. 3) a good electrostatic and steric complementarity can be observed between each individual fragment and its corresponding binding motif (Fig. 3D and E). In fact, the *P*Cho and phosphate moieties of the docked model nicely superimpose with the bound choline and sulfate molecules found in the crystal structure of CbpL_CBM_ (Fig. 3D).

The computational model of the complex of a teichoic acid (TA) bound to the CbpL_CBM_ was built by means of iterative cycles of docking and restrained MD simulations. The modeled TA represents two RU of the WTA/LTA polymer^28^, consisting of five concatenated modified sugar moieties (namely (6-O-*P*Cho)-α-GalpNAc, (6-O-*P*Cho)-β-GalpNAc, ribitol-5-*P*-β-Glcp, and α-AATGalp). In the refined solution (Fig. 3E and F), the *P*Cho moieties of (6-O-*P*Cho)-α-GalpNAc occupy the NCS whereas the *P*Cho of the (6-O-*P*Cho)-β-GalpNAc) subunit is lodged in a CS. In such a conformation, the ribitol-5-phosphate is hydrogen bonded (Fig. 3E) to the side chains of the underlined Tyr residue within the motifs –WY**Y**L– (Tyr105/145/195) from the long repeats, the carboxylate of Asp149/189/229/265 and with hydroxyl groups of Ser151/191/231 (Fig. 3E and F). The C4-amino group of AATGalp establishes a H-bridge with Gln residues within the motifs –W**Q**GNY– (Gln118/158/198) from the short repeats (Fig. 3F and Fig. S7). Concomitantly, the β-Glc residue that is disposed covering the Gln118/158/198/238 residue is hydrogen bonded through OH3′ to carbonyl oxygen of Trp157/197/237. In such a disposition, β-Glc exposes its hydroxyl groups to the bulk solvent, which is compatible with the reported acylation of its OH2′ and OH4′ groups with D-Ala residues^28^. Finally, the C4-amino group of the unusual AATGalp is hydrogen bonded to the carboxamide oxygen of Gln158/198/238 (Fig. 3F). Due both to the repetitive pattern of CS and NCS sites along the CbpL_CBM_ and the conservation of critical CbpL residues involved in TA recognition, up to four RU of TA can wrap around the CBM in a vine-like fashion.

### NMR structure of the Excalibur domain of CbpL

Due to the problems in crystallization of the full-length CbpL structural determination of the isolated Excalibur domain was conducted by using multidimensional NMR spectroscopy (Table 2). The three-dimensional structure of the Excalibur domain plus two residues of the linker (residues Glu27-Lys70) was determined in solution as described in the experimental section. Backbone and side-chain assignments of the Excalibur domain were obtained using standard 2D techniques. In the absence of Ca^2+^ in the medium the NMR spectra showed that the protein exists as a random coil or as an unfolded polypeptide. Addition of Ca^2+^ dramatically changed the distribution and the dispersion of the NMR signals (Fig. S9) indicating that the protein was folded upon Ca^2+^ binding. This fact reveals a drastic conformational change in the presence of Ca^2+^ that is compatible with the folding of the polypeptide chain as a consequence of the existence of a conserved DxDxDGxxCE motif in Excalibur^19^ with high sequence similarity to the Ca^2+^-binding loops of the calmodulin-like EF-hand domains. For this reason, we have structurally characterized the folded entity in the presence of saturated concentrations of Ca^2+^.

**Figure 4:**
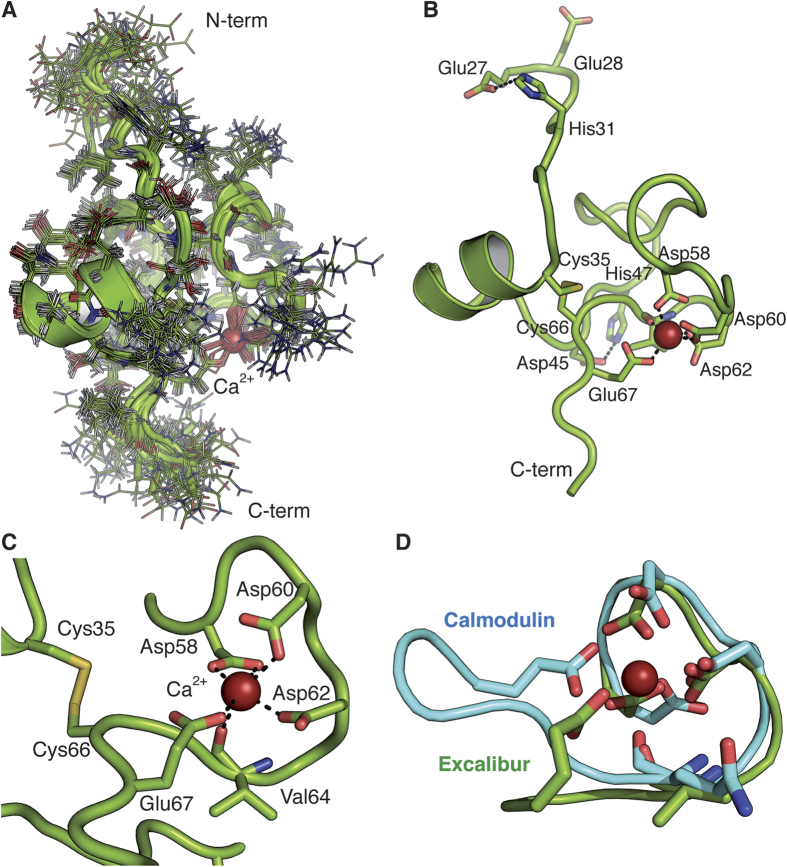
Structure of the Excalibur domain determined by NMR. (**A**) Superposition of the 20 low energy conformers of the Excalibur domain determined by NMR. Ca^2+^ is depicted in red sphere. (**B**) Ribbon representation of one Excalibur conformer showing its main structural features. (**C**) Ca^2+^-binding loop architecture and localization of the disulfide bridge. (**D**) Backbone superimposition between the Ca^2+^-binding loop of the Excalibur domain and calmodulin (PDB code 1CLL) with a rmsd_backbone_ = 0.69 Å.

The superposition of the 20 low energy conformers of the Excalibur domain determined by NMR is shown in Fig. 4A. The structure has a disordered N-terminal short tail (from Glu27 to His31), a globular packed domain containing a short α-helical region (residues 35–40) plus a Ca^2+^-binding site (residues 58–67) and, finally, a disordered C-terminal region (residues 68–70) at the beginning of the linker (Fig. 4B). The global fold of the domain shows an intricate globular shape (16 × 20 × 27 Å). The polypeptide chain shows a complete lack of secondary structure except for a 6 residue-long helical region (^35^CKEAWAN^40^) located near the N-terminus. A search for proteins structurally related to the Excalibur domain with the DALI server^27^ returned no statistically significant matches. All the residues in the ^58^DxDxDGxxCE^67^ motif, which were predicted as characteristic for a new type of extracellular Ca^2+^-binding regions in bacteria^19^, are indeed involved in Ca^2+^ binding. The side chains of Asp58, Asp60, Asp62 and Glu67 interact with Ca^2+^ in a canonical disposition (Fig. 4B and C). The two cysteine residues present in all the Excalibur domains establish a disulfide bridge that connects the short α-helical region with the Ca^2+^-binding site (Fig. 4B). This Ca^2+^-binding motif of the Excalibur domain is strikingly similar to the Ca^2+^-binding loop of the calmodulin-like EF-hand domains (rmsd of 0.69 Å for the Cα backbone of calmodulin; PDB code 1CLL) (Fig. 4D). Indeed, a comparison of Excalibur and EF-hand consensus sequences demonstrates conservation in the former of the three Ca^2+^-binding Asp residues of the EF-hands and compatibility of the surrounding residues. In this motif, the Ca^2+^ ion binds in a pentagonal bipyramidal fashion with the side-chains of three turn residues, typically aspartates, acting as monodentate ligands. A further chain, typically Glu, is a bidentate Ca^2+^ ligand while the main chain carboxyl group of another residue (Val64 in Excalibur) and water molecules complete the coordination of the Ca^2+^ ion.

**Figure 5:**
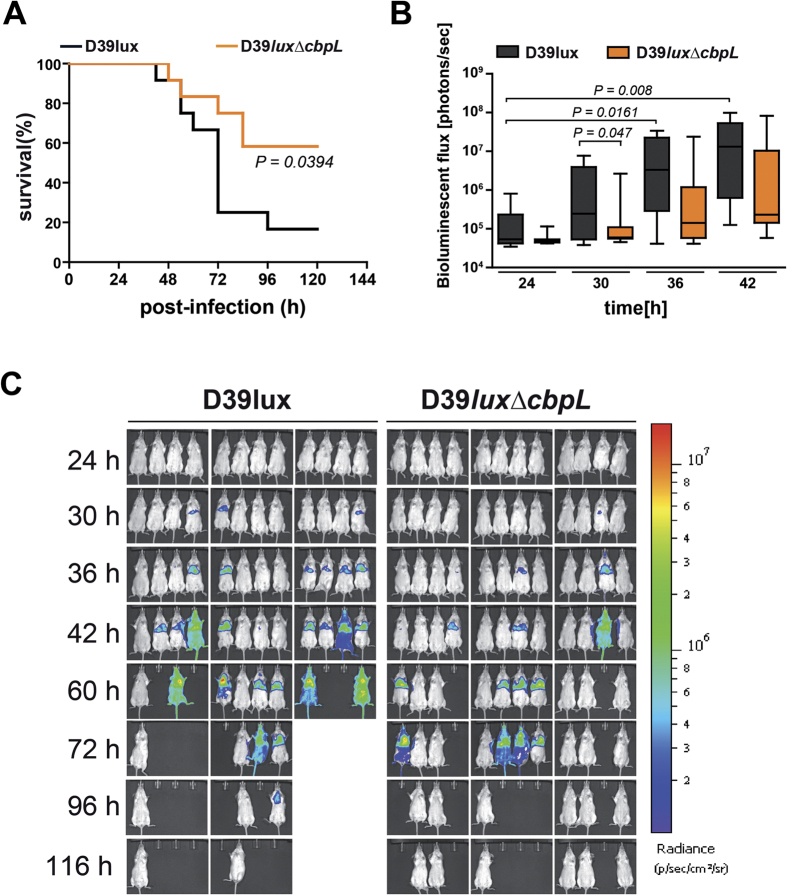
Loss of function of CbpL attenuates pneumococci in the acute pneumonia infection model. **(A)** Kaplan-Meier survival plot of CD-1 mice infected with D39 wild-type and CbpL-deficient pneumococci. Mice (n = 12) were intranasally infected with 2.0 × 10^7^ CFU of *S. pneumoniae* D39 wild-type or its isogenic ∆*cbpL*-mutant. (**B**) and (**C**) Bioluminescent optical imaging of pneumococcal dissemination after intranasal infection of CD-1 mice (n = 12). Transmigration of bioluminescent D39*lux* and D39*lux*∆*cbpL* into the lung or blood was analyzed at indicated time points by measuring of the luminescence intensity using the IVIS^®^ Spectrum System. The bioluminescent flux of grouped mice is represented in the box whiskers graph (**B**).

Structurally, the Excalibur domain shows some other interesting characteristics that can be crucial for its function or interaction with binding partners: (i) despite its small size (44 residues), there is a high number of hydrophobic (20.45%) and aromatic residues (9%) that in most cases are tightly packed although four of them (Trp39, Tyr43, His47 and Tyr53) are quite exposed to the solvent suggesting some role for interactions (Fig. S10A); (ii) the Excalibur domain has a characteristic distribution of the molecular electrostatic potential displaying a positively-charged patch on one face a negatively-charged patch on the other (Fig. S10B); (iii) the domain structure is further stabilized by several hydrogen bonds and a salt bridge interaction (Asp45-His47) (Fig. 5B); (iv) four glycine residues (Gly39, Gly46, Gly49 and Gly60) that are highly conserved within the Excalibur domains of different bacterial species^19^ are located in turn regions preceding a kink of the backbone where any other residue would be sterically hindered, pointing to the importance of these residues in allowing folding of the Excalibur domain upon Ca^2+^ binding; (v) the flexibility of the N-terminal segment (^27^EENIH^31^) of the Excalibur domain is a striking feature worth mentioning. This segment presents strong disorder in the 20 low-energy conformers that seems to act as a flexible antenna of the Excalibur domain exposing two negatively charged residues to the medium (Fig. 4B). In some of the conformers, a salt bridge interaction is formed between Glu27 and His31.

### Loss of function of CbpL attenuates pneumococcal virulence

CbpL-deficient mutants showed a similar growth behavior in chemically defined media (Fig. S11) compared to the CbpL-expressing strain. In an earlier study CbpL was shown to interact with collagens, elastin and C-reactive protein, suggesting that CbpL contributes to the pathogen-host interaction of pneumococci^29^. To decipher whether CbpL is involved in colonization and lung infection, the acute pneumonia mouse model of infection was applied. Outbred CD-1 mice (n = 12) were infected intranasally with 2.0 × 10^7^ bioluminescent *S. pneumoniae* wild-type strain D39 (D39*lux*) or its isogenic mutant strain lacking CbpL (D39*lux*Δ*cbpL*). The dissemination of pneumococci was monitored by real-time bioimaging. Wild-type infected mice started to show first signs of a lung infection 30 h post-infection with a severe progression leading to sepsis 42 h post infection (Fig. 5C).

The infection process in mice infected with D39*lux*Δ*cbpL* was appreciably delayed as indicated by retarded increase in bioluminescence (Fig. 5C and B). In addition, the survival rate of mice infected with CbpL-deficient pneumococci is significantly higher compared to mice infected with D39*lux* bacteria (Fig. 5A). Taken together these data demonstrate a critical role of CbpL in pneumococcal lung infections and spread of pneumococci from the nasopharynx into the lungs and blood.

### The deficiency of CbpL is associated with a higher pneumococcal phagocytosis rate

**Figure 6:**
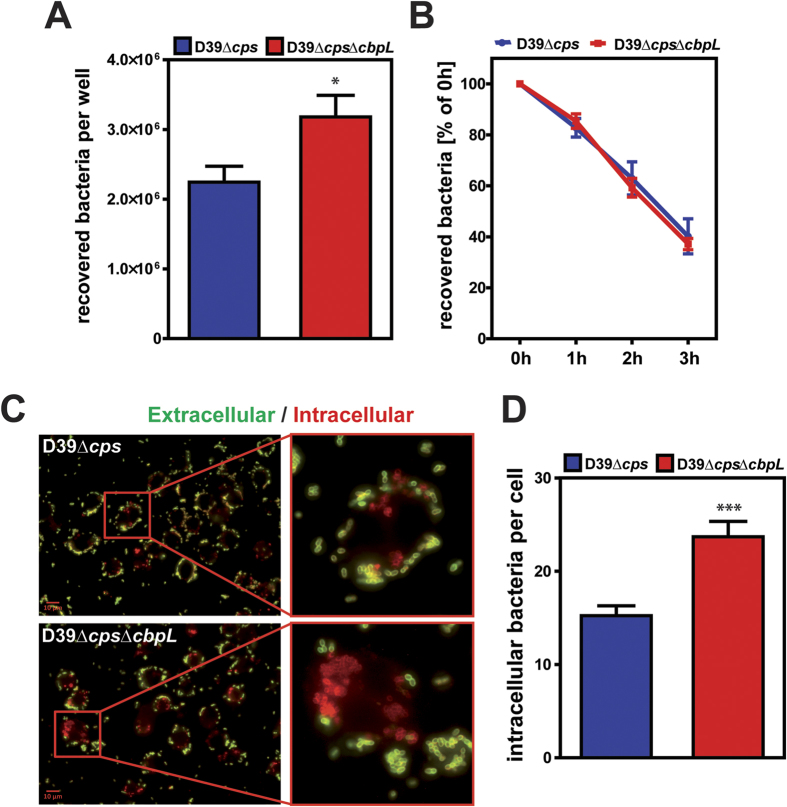
Effect of CbpL deficiency on uptake of *S. pneumoniae* D39Δ*cps* by professional phagocytes. (**A**) J774 murine macrophages were infected for 30 min with non-encapsulated D39Δ*cps* or its isogenic *cbpL*-mutant. The multiplicity of infection was 50 bacteria per cell. The recovery of intracellular pneumococcal survivors was quantified by applying the antibiotic protection and plating the bacteria on blood agar plates. Experiments were done at least three times in triplicate and data represent the mean of recovered bacteria per well ± SEM. *P < 0.05. (**B**) Intracellular survival of pneumococci. Infected J774 cells were lysed either immediately after 30 min infection and post antibiotics treatment (t = 0) or one, two or three hours post antibiotic treatment. Experiments were done at least three times in duplicate and data represent the mean of recovered bacteria per well ± SEM. (**C**) Immunofluorescence microscopy of pneumococci attached (green) to J774 macrophages and phagocytized, intracellular pneumococci (red) 30 min post infection. Attached bacteria were stained with goat anti-rabbit Alexa Fluor 488 (green), while intracellular bacteria were stained with goat anti-rabbit Alexa Fluor 568 (red) after using rabbit anti-pneumococcal polyclonal antibodies as the first antibody. Intracellular bacteria were stained post Triton X-100 treatment to permeabilize macrophages. (**D**) Number of phagozytosed pneumococci (mean ± SEM) per macrophage as determined by counting intracellular pneumococci in at least 50 macrophages of double immunofluorescence microscopic samples. ***P < 0.001.

CbpL-deficient pneumococci are attenuated under *in vivo* conditions. Lung infections are characterized by an infiltration of leukocytes and macrophages, hence, the demonstrated loss of virulence can be attributed to a lower resistance against phagocytosis. The impact of CbpL on uptake by professional phagocytes and the capacity of pneumococci to survive intracellularly were therefore assessed by infecting murine macrophages J774A.1 with D39Δ*cps* or its isogenic *cbpL*-mutant D39Δ*cps*Δ*cbpL*. The number of recovered survivors was determined by killing extracellular bacteria 30 min post-infection by antibiotic treatment. Colony forming units of recovered intracellular pneumococci were determined by plating the infected cells on blood agar plates. The enumeration revealed a significantly higher number of Δ*cbpL-*mutants compared to the parental strain D39Δ*cps* (Fig. 6A). The recovery of intracellular pneumococci at different time points post-infection and after killing extracellular bacteria 30 min after infection suggested that the intracellular survival rate of CbpL-deficient pneumococci is similar to the parental strain expressing CbpL (Fig. 6B). To visualize and quantify the efficiency and differences of bacterial uptake, pneumococci bound to macrophages (green) or located intracellularly (red) were labeled by double immunofluorescence staining. The immunofluorescence microscopy suggested a higher number of CbpL-deficient pneumococci inside of macrophages compared to the isogenic parental strain (Fig. 6C and Fig. S12). Indeed, enumeration of intracellular pneumococci revealed a significant higher number of intracellular *cbpL*-mutants compared to the isogenic D39 Δ*cps* (Fig. 6D), suggesting that CbpL contributes to resistance against phagocytosis. Taken together, the antibiotic protection assays and immunofluorescence microscopy suggest that CbpL prevents uptake by phagocytes but does not modulate the intracellular fate as there was no difference in the ability of the *cbpL*-mutants to survive intracellularly compared to the parental strain. In other words, the higher rate of CbpL-deficient uptake by macrophages promotes elimination of pneumococci in infected host areas.

## Discussion

The pneumococcal cell wall contains the peptidoglycan layer with covalently attached WTA, and membrane-bound LTA that, only in pneumococci, present unusual complex RUs of identical chemical structure^30^. We provide here insights into the structure and function of CbpL, an enigmatic multi-modular protein presenting an Excalibur domain, a unique CBM in the middle and a C-terminal domain with homology to lipoproteins of temperate phages (Ltp). We disclose here, for the first time, the structure of the Excalibur domain. This domain is unfolded in the absence of Ca^2+^ and folded in a compact globular fashion upon Ca^2+^ binding. The five first residues at the beginning of the Excalibur are disordered, presenting some charged resides (two Glu and one His residues) that are fully exposed.

The CBM allows anchoring of CBPs to the pneumococcal cell wall through non-covalent binding of PCho moieties of WTA and LTA. RUs of both WTA and LTA contain the same pseudo-pentasaccharide building blocks^31^. The terminal RU (comprising the Forssman antigenic disaccharide (α-D-GalpNAc-(1–3)-β-DGalpNAc-(1–1)-ribitol)) can occur with or without 6-O-PCho substitutions on both GalpNAc and each RU in TA can also be mono-PCho-substituted (lacking the *P*Cho at β-GalpNAc)^31^. The chain lengths of the WTA and LTA in pneumococci seem to be very similar and consistent among strains, the most common being 6–7 RUs. Independently of the degree of biosynthetic PCho substitution in TA, it has been reported that pneumococcal phosphorylcholine esterase (named Pce or CbpE) also modifies PCho decoration by releasing 15–30% of *P*Cho residues from TA^32^.

The three-dimensional structure of the CbpL_CBM_, while following the general superhelical fold found in other CBPs, presents a unique combination of non-consensus repeats building a total of eight choline-binding sites, four of them having the canonical stabilization of choline by three aromatic residues, and the other four sites presenting a non-canonical arrangement formed by four aromatic residues each. Surprisingly, an alternate distribution of canonical and non-canonical sites is observed along CbpL_CBM_. Molecular dynamics calculations provide explanation for such unprecedented finding: the CbpL_CBM_ stabilizes the TA unit in an spiral, vine-like manner allowing simultaneous binding of fully substituted GalpNAc units by two phosphoryl choline moieties. Therefore CbpL would be strongly attached to the cell wall in those places in which WTA/LTA presents two PCho moieties per unit. Complete binding of all the available sites in CbpL_CBM_ would imply interaction with four units of WTA/LTA polymers.

The available structures of several CBPs indicate the existence of differences in the number and nature the of choline-binding sites in the CBP family, suggesting the existence of specific choline-binding site combinations which could act as a recognition pattern, providing an specific affinity or location to each protein within the wall of *S. pneumoniae*. Such is the case of LytB, located at the poles of the cell^33^, or CbpD and LytA that are predominantly targeted at the cell separation septum^5^,^34^. Other proteins, however, are uniformly located across the wall, as is the case of CbpF^16^. Our structural and bioinformatics models indicate that CbpL could preferentially recognize those TA having Bi-*P*Cho-substituted TA. It is not known at present whether these fully-PCho-substituted TA are distributed all over the cell wall or whether they are restricted to preferential locations. Our immunofluorescence microscopy assays, however, reveals that CbpL is non-homogeneously distributed on the cell wall, thus pointing to specific locations for this protein.

Besides the Excalibur domain and the choline-binding module, CbpL presents a C-terminal domain homologous of the Ltp family (pfam07553). Ltp proteins are characterized by (i) a repeat of two helix-turn-helix domains, (ii) an acidic pI-value, and (iii) a membrane-anchoring N-terminal region^20^. On the basis of the high sequence similarity (66.7%) between the Ltp domain of CbpL and the same domain from *Streptococcus thermophilus* phage TP-J34, a computational model was prepared for CbpL_Ltp_ domain (see methods). The model for the CbpL_Ltp_ domain (Fig. S13) presents three α helices following the predicted HTH motif. The amino acid composition of the CbpL_Ltp_ domain also presents an acidic character (pI 5.99) in agreement with Ltp family proteins. However, while all the reported members of the Ltp family present a Cys residue at N-term that anchors the lipoprotein to the membrane^20^ no such residue (nor lipobox motif (LVI)(ASTVI)(GAS)C) is present in CbpL_Ltp_. Our experimental results also confirm that CbpL is not a lipoprotein. This unique feature within the Ltp family points to a specific target for CbpL. In the case of phage TP-J34, it has been reported that Ltp protein remains anchored to the membrane to block DNA-injection by other phage competitors during the superinfection exclusion process. Experimental data supports that inhibition is produced by a direct protein-protein interaction between the Ltp and the phage’s tape measure protein^20^. Therefore, in CbpL a similar interaction with another protein and/or cell-wall component could be expected through the Ltp domain.

**Figure 7:**
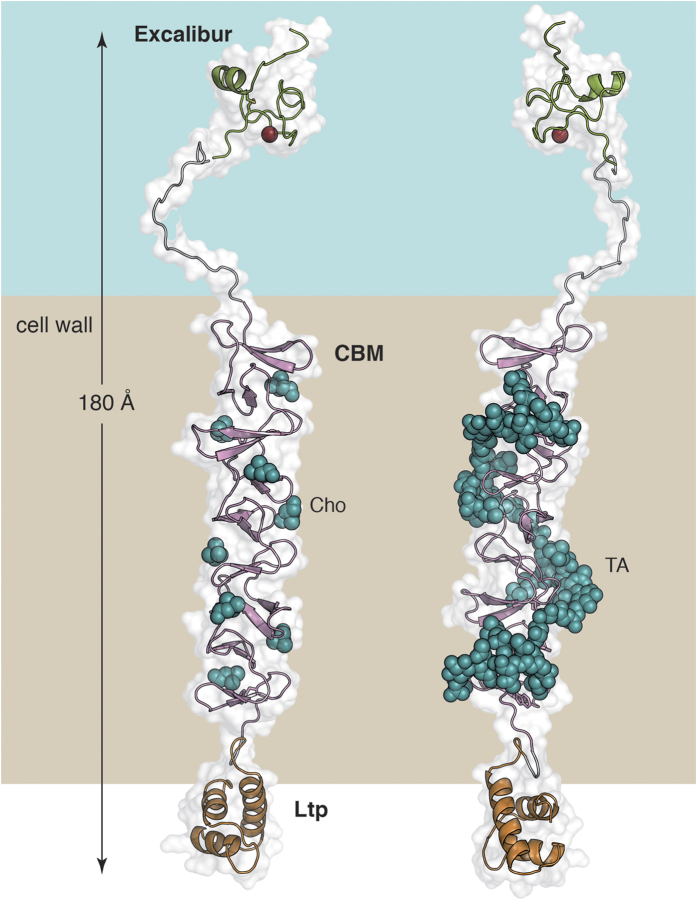
Three-dimensional model for the full-length CbpL. (Left) Each domain is colored (Excalibur in green, choline-binding module in pink and Ltp domain in orange). Disordered linkers colored in grey. Choline molecules depicted as green spheres. (Right) CbpL with a teichoic acid (green spheres) composed of four units attached. Each of the four TA units in the model is fully substituted with two phosphoryl choline moieties.

CbpL presents two linker regions, one of them connecting the Excalibur domain with the CBM, and the second one connecting the CBM with the Ltp domain. According to PrDOS (Protein DisOrder prediction System)^35^ both linker regions are predicted as intrinsically disordered. The first linker region is very long (27 amino acids) and presents a high pI-value (10.48) provided by four Lys residues. The second linker is a 14 amino acids-long region very rich in serine residues (six). Both linkers seem to act as spacers to position the different domains of CbpL at specific positions in the pneumococcal cell wall. Considering both our structural information for the Excalibur and CbpL_CBM_ provided by NMR and X-ray crystallography, respectively, and the computational model for the Ltp domain, a structural model for the full-length CbpL can be envisaged (Fig. 7). CbpL presents a very elongated shape of >180 Å-long. Considering the large number of choline attachments in CBM, it is expected that CbpL would remain strongly anchored to the peptidoglycan layer, exposing the Excalibur domain to the capsule or the extracellular medium.

Little is known about the biological function of CbpL. A previous study with different pneumococcal surface proteins revealed the capacity of CbpL to interact with collagen, elastin and C-reactive protein^29^ suggesting a role in adhesion or immune evasion during the *S. pneumoniae* infection process. In this work, we have deciphered the impact of CbpL on pneumococcal pathogenesis and resistance against phagocytosis. Our studies indicated that CbpL plays a critical role both in pneumococcal lung infections and in transmigration from nasopharynx to the lungs and blood, therefore pointing to a direct role of CbpL in host-pathogen interactions. In the acute pneumonia mouse infection model the CbpL-deficiency attenuated pneumococci and consequently, transmigration in the lungs as well as the ability to reach the bloodstream was substantially impaired. In agreement with the observed *in vivo* attenuation, assays with macrophages proved that CbpL contributes to resistance against phagocytosis, therefore confirming its role in subverting immune response and recognition of this important human pathogen. Nevertheless, the individual role of the CbpL modules remains unsolved. It can be anticipated that the CBM plays no direct role in virulence because of its implication in protein attachment to the bacterial cell surface. In contrast, the Excalibur and/or the Ltp_Lipoprotein domain may have important functions in protein-protein interactions leading to recognition of soluble host proteins or receptors. Even so one might speculate that these domains have the potential to modulate the immune response by yet unknown mechanisms. This would fit to the idea that CBPs contribute to pneumococcal pathogenesis or immune evasion via sequestering of serum or matrix proteins or interacting directly with cellular receptors. Regulation of CBPs is not very well known. There are some intriguing studies showing that the choline-binding protein PspC (CbpA) is regulated by the TCS06^36^. Gene expression of *cbpL* is currently unknown and its expression could be regulated by any of the pneumococcal TCS and maybe co-regulated with *zmpB* expression, which has been shown to be regulated by the response regulator of TCS09^37^.

## Final Remarks

The here provided, structural and functional characterization of this multi-modular protein reveals a combination of unique non-enzymatic modules directly linked with the pneumococcal ability to colonize and subvert immune recognition. Our integrated/multidimensional study provides a sound approach into the complexity of the role performed by CbpL in the pneumococcal lung infection, and points that redundancy of surface adhesins is needed for pneumococci to sequentially interact with host cells during the invasive disease.

## Materials and Methods

### Bacterial strains, culture conditions, and transformation techniques

*E. coli* strains and *S. pneumoniae* genotypes used in this study are listed in Table S1. Bacteria were cultured and transformed as described^38^. Briefly, *E. coli* was cultured on Luria-Bertani (LB) plates or in LB broth, which were supplemented with ampicillin (100 μg/mL) erythromycin (250 μg/mL), and/or kanamycin (50 μg/mL). For Labeled ^13^C/^15^N protein used in NMR studies, cells were grown in LB broth and, previous to protein induction, they were changed to a modified M9 minimal medium containing isotopically labelled ^13^C-U-glucose (4 g/L) and ^15^NH_4_Cl (1 g/L) as sole carbon and nitrogen sources^39^. Transformation of *E. coli* was carried out with CaCl_2_-treated competent cells and pneumococci were transformed as described^40^ using competence-stimulating peptide-1 (or competence-stimulating peptide-2 for TIGR4-strains). *S. pneumoniae* and isogenic mutants were grown on blood agar plates (Oxoid, Germany), cultured in Todd-Hewitt broth (Oxoid, Basingstoke, England) supplemented with 0.5% yeast extract (THY; Roth, Karlsruhe, Germany) or in chemically defined medium RPMImodi^41^. Cultivation of pneumococci was conducted at 37 °C and 5% CO_2_, and liquid cultures were cultivated to mid-log phase (A_600_ 0.35 to 0.4) without agitation. Mutants were cultivated in the presence of the appropriate antibiotics: erythromycin (5 μg/mL), and/or kanamycin (50 or 150 μg/mL).

### Primers and molecular techniques

Primers that were used in this study and plasmids used for the mutagenesis and recombinant protein expression are listed in Tables S1 and S2. Isolation and purification of genomic pneumococcal DNA was performed using the QIAGEN Genomic Tip 100/G (Qiagen, Hilden, Germany) according to the manufacturer’s instructions with slight modifications described earlier^42^. DNA amplifications needed for mutagenesis were carried out by PCR. To amplify pneumococcal DNA by PCR the *Taq* DNA polymerase (New England Biolabs, Frankfurt, Germany) was used and the reactions were subjected to 30 cycles of denaturation at 94 °C, primer annealing for 30 sec, and elongation at 72 °C. The annealing temperature depended on the primers and extension time on the length of PCR product. For expression cloning the proofreading *Pfu* polymerase was used as specified by the manufacturer (Stratagene, LaJolla, U.S.). Oligonucleotides were synthesized by Eurofins MWG Operon (Germany). PCR products were purified with the PCR DNA purification kit (Qiagen, Hilden, Germany) and plasmids were isolated and purified with the Qiaprep Spin Midi or Maxiprep Kit (Qiagen, Hilden, Germany). The integrity of the DNA was confirmed by sequencing (Eurofins MWG Operon).

### Construction of pneumococcal mutants

To construct pneumococcal mutants in D39*lux*^43^ and D39Δ*cps* the insertion-deletion mutagenesis strategy was used^42^. To generate *cbpL-*mutants the loci of the gene and their upstream and downstream flanking sequences were PCR amplified from *S. pneumoniae* serotype 2 strain D39 (NCTC7466) and TIGR4, respectively, genomic DNA as template and using primers cbpL_632/cbpL_409 and cbpL_408/cbpL_409 (S1 Table).

PCR products were directly cloned into pGEM^®^-T Easy according to the manufacturer´s instructions (Promega, Madison, U.S.) and transformed into *E. coli* DH5α competent cells. The recombinant plasmids (p716 and p562) containing the DNA-inserts of interest were isolated, purified and used as template for the inverse PCR reaction with primer pairs possessing incorporated restriction sites (cbpL_408/cbpL407). The deleted *cbpL* sequences in the plasmids were replaced with the *ermB* gene cassette PCR amplified from plasmid pE89 using the primer combination ermB_105/ermB_106 (S2 Table). The integrity of the DNA-inserts was confirmed by PCR, restriction analysis, and DNA sequencing (data not shown). The plasmids p717 (for D39) and p569 (for TIGR4) were used to transform the corresponding pneumococci as described^40^ and mutants were selected by the addition of erythromycin (5 μg/mL).

To verify the stability of the mutants they were cultivated at least two times in liquid culture without antibiotics before identical culture volumes were spread on blood agar plates with or without erythromycin. The mutants were considered to be stable (not shown).

### Expression and cloning of CbpL

The *cbpL* gene sequence lacking the signal sequence (*sp_0667*; nt 79 to nt 996; residues 27 to 332) was amplified by PCR with the primer pair cbpL_461/cbpL_462 and *S. pneumoniae* TIGR4 chromosomal DNA as template. Primers had *Nhe*I/*Sac*I restriction sites (Primers are listed in S2 Table). The PCR product was cloned into the similarly digested pTP1 expression vector. In pTP1 the thrombin cleavage site of the pET28b vector (Novagen) is replaced by the TEV protease cleavage of the pProEx HTa vector (Life Technologies, Darmstadt, Germany) and also the multiple cloning site was slightly modified^38^. A further expression CbpL expression clone was constructed by amplifying the *cbpL* using the primer pair cbpL_598/cbpL_462 (with incorporated *Nco*I/*Bam*HI restriction sites) and TIGR4 chromosomal DNA as template. The digested PCR product was cloned into similarly digested pET28a (Novagen). The plasmids were transformed into *E. coli* BL21 (DE3) resulting in plasmids p630 (His_6_-tag protein) and p718 (referred to as untagged protein). The Excalibur domain of CbpL including Glu27 to Asn70 was amplified using primers cbpL_461 and cbpL_1156 and cloned into pTP1 vector to allow the fusion of the N-terminal His_6_-tag followed by the TEV cleavage site. This resulted in plasmid p1070. The Excalibur-CBM fragment of CbpL ranging from Glu27 to Glu270 was amplified with primers cbpL_461/cbpL_1321 and similarly to the other *cbpL* sequences cloned into pTP1 resulting in plasmid p1025. Integrity of insert DNA was confirmed by DNA sequencing.

### Protein purification and immunoblotting

For protein production the recombinant *E. coli* BL21 (DE3) harboring the plasmids p630, p718 or p1070 were cultured in LB broth, supplemented with kanamycin (50 μg/mL), and grown to an A_600_ of 0.5 to 0.7 at 30 °C. Protein expression was then induced with 1 mM IPTG (isopropyl-β-D-1-thiogalactopyranoside) and cultivation was continued for 4 h. The His_6_-tagged proteins were purified by affinity chromatography using His Trap™ HP Ni*-*NTA columns (1 mL; GE Healthcare, Chalfont St Giles, UK) and the ÄKTApurifier liquid chromatography system (GE Healthcare) according to the instructions of the manufacturers (Fig. S14). The His_6_-tag was cleaved off by TEV digestion overnight on ice followed by a further purification step using the His Trap™ HP column. The untagged proteins were collected in the flow through. The purified CbpL-proteins (without His_6_-tag) were dialyzed against 20 mM Tris-HCl (pH 8.0) and concentrated to 1–5 mg/mL using Vivaspin Ultra concentrators (Sartorius, Göttingen, Germany).

To purify the untagged rCbpL protein *E. coli* BL21(DE3) cells were resuspended after protein induction (overnight at 16 °C) in 10 mL of 20 mM sodium phosphate pH 6.9 per litre of culture, supplemented with cOmpleteTM EDTA-free protease inhibitor cocktail (Roche). Cell lysis was performed by sonication on ice (15 pulses of 15 s each) followed by 50 min centrifugation at 40000 × g. The supernatant was incubated with streptomycin sulphate (Sigma-Aldrich) for 20 min at 4 °C, followed by a further centrifugation step of 20 min at 20000 × g to remove the nucleic acids from the sample. The soluble fraction containing rCbpL was added to a 5 mL HiTrap Q FF column (GE Healthcare) previously equilibrated with 20 mM sodium phosphate pH 6.9 (buffer A). After binding of rCbpL the column was washed with 5 volumes of buffer A, 10 volumes of buffer B (20 mM sodium phosphate pH 6.9; 1 M NaCl) and 5 volumes of buffer C (20 mM sodium phosphate pH 6.9; 150 mM NaCl). Finally, pure rCbpL was eluted with a gradient of choline chloride (Sigma-Aldrich), reaching a maximum concentration of 0.5 M.

Purity of the proteins was analyzed after sodium dodecyl sulfate polyacrylamide gel electrophoresis (SDS-PAGE) by both Coomassie brilliant-blue (CBB) staining and immunoblotting. Detection of CbpL proteins (or *S. pneumoniae* CbpL-proteins) was carried out by using polyclonal mouse anti-CbpL antisera (1:1000). After SDS-PAGE separation of recombinant proteins or bacterial lysates, proteins were transferred to a nitrocellulose membrane by using a semidry blotting system (Bio-Rad, Laboratories, Munich, Germany). The membrane was blocked with 5% skim milk (Roth, Karlsruhe, Germany). As secondary antibodies goat anti-mouse Ig peroxidase conjugate (Dianova; 1:5000) were used and binding activity was detected by enhanced chemiluminescence (Luminol and p-coumaric acid, Roth).

### Crystallization of CbpL_CBM_

Prior to setting up crystallization assays, CbpL buffer was exchanged to 10 mM Tris-HCl pH 7.5 and 140 mM choline chloride by dialysis. First crystallization screenings were performed by high-throughput techniques in a NanoDrop robot and Innovadyne SD-2 microplates (Innovadyne Technologies Inc.), screening PACT Suite and JCSG Suite (Qiagen), JBScreen Classic 1–4 and 6 (Jena Bioscience) and Crystal Screen, Crystal Screen 2 and Index HT (Hampton Research). Positive conditions were optimized by hanging-drop vapour diffusion method by mixing 1 μL of protein solution and 1 μL of precipitant solution, equilibrated against 500 μL of precipitant solution. Best crystals were obtained in a crystallization condition containing 1.6 M ammonium sulphate and 0.5 M LiCl, at a protein concentration of 40.8 mg/mL.

### Data collection and structural determination of CbpL_CBM_

Prior to data collection, crystals were cryoprotected in the precipitant solution supplemented with 20% (v/v) glycerol. Diffraction data was collected in beamline X06SA (PXI) at the Swiss Light Source (SLS, Villigen, Switzerland), using the Pilatus 6 M detector. Crystals diffracted up to 1.7 Å resolution and belonged to the P2_1_ space group, being the unit cell parameters *a* = 30.85 Å, *b* = 42.79 Å, *c* = 70.62 Å, α = γ = 90°, β = 101.06°. The collected datasets were processed with XDS^44^ and Aimless^45^. A truncated single monomer of the choline-binding module of CbpL (named CbpL_CBM_) was found in the asymmetric unit, yielding a Matthews coefficient of 2.18 Å^3^/Da^46^ and a solvent content of 43.6%. To assess the fragment identity, a peptide mass fingerprint over dissolved crystals was performed.

Several partial fragments of the choline-binding domain of CbpF (PDB code 2V04) were used as initial models for molecular replacement in Phaser^47^,^48^. Refinement and manual model building of the CbpL_CBM_ were performed with Phenix^49^ and Coot^50^ respectively. The stereochemistry of the final model was checked by MolProbity^51^. The quality of the electron density map allowed to build the structure of CbpL_CBM_ from Thr92 to Ala273, showing eight choline binding sites and a choline molecule stabilized in each of them. Data collection and processing statistics are shown in Table 1. The atomic coordinates of CbpL_CBM_ have been deposited in the Protein Data Bank under code 4CNL.

### NMR samples and assignment

Typically, NMR samples contained up to 0.5 mM of protein unlabelled and ^13^C, ^15^N labelled) were prepared in 90% H_2_O/10% D_2_O at pH 7 and 5.5 (uncorrected for deuterium isotope effects) with different concentrations of CaCl_2_ (0, 1 and 3 mM). Sodium-4,4-dimethyl-4-silapentane-1-sulfonate (DSS) was used as internal ^1^H chemical shift reference. 13C and 15N chemical shifts were indirectly referenced by using the IUPAC-IUB recommended chemical shift ratios^52^. All spectra were recorded on a Bruker AV800 spectrometer equipped with a cryoprobe TCI at 25 and 35 °C. The assignment of ^1^H, ^13^C and ^15^N resonances was achieved using a standard suite of heteronuclear 2D and triple resonance 3D spectra: ^1^H-^1^H TOCSY (60 ms mixing time), ^1^H-^1^H NOESY (50 and 80 ms mixing times), ^1^H-^15^N HSQC, ^1^H-^13^C HSQC, HNCO, HNCOi, HNCA, HNCAi, HNCACB, CBCA(CO)NH, H(CCCO)NH, (H)CC(CO)HN, HC(C)H-TOCSY (15 ms mixing time), (H)CCH-TOCSY (15 ms mixing time), as previously described^52^. As an additional validation, two 3D experiments H(NCOCA)NH and (H)N(COCA)NH^53^,^54^, NMR experiment were recorded and analyzed. Spectra were processed with Topspin (Bruker Biospin, Karlsruhe, Germany) or NMRPipe^55^ and analysed with SPARKY^56^ and NMRView^57^. Backbone and side chain ^1^H, ^13^C and ^15^N chemical shifts were assigned following conventional strategies, the resonance list is complete and has been deposited in the BioMagResBank (http://www.bmrb.wisc.edu/) under accession number 30064.

### NMR structure calculation

The two Cys residues in this domain are suggestive of the existence of a disulphide bridge. It has been reported that they are entirely conserved in the extracellular protein family mentioned above. It may be that this structural constraint substitutes for the larger domain framework of EF-hand containing proteins in orienting the ends of the Ca^2+^ binding loop correctly for metal binding. First, we calculated the solution structure with all the NMR restraints without imposing S-S restrictions. In the resulting structure, the SH atoms were located at the proper distance and orientation for S-S bond formation, showing that the covalent bond is fully compatible with the NMR restraints. Then, with this information we proceeded to calculate the final structure with the explicit disulphide bond. The solution structure of the Excalibur domain was calculated with the program Cyana v2.1^54^ based on experimental NOE-derived distance constraints and Talos+-derived dihedral constraints^58^ following the standard 7-cycle iterative process and a final annealing using the list of restraints obtained in the last cycle. 100 structures were generated. Constraint files included 564 upper distance restraints for protons (268 intra, 118 medium-range, 178 long-range) 12 restraints for the S-S bond, and 8 for Ca^2+^ binding, complemented with 58 φ and ψ restrictions. The 20 conformers with the lowest target function values were selected for further refinement and finally minimized with Amber9 software^59^ using the Gibbs-Boltzmann continuum solvation model. The structure has been deposited in the Protein Data Bank under the accession number 5J8T. The structural ensembles were visualized and examined using MolMol^9^ and PyMOL^60^.

### Molecular Dynamics and docking calculations

The geometries of individual TA subunits capped with a methyl group were optimized using program Gaussian 09 (Gaussian, Inc., Wallingford, CT). Charges were then assigned to individual atoms by fitting the quantum mechanically calculated (RHF/6–31G*//RHF/3–21G*) molecular electrostatic potential to a point charge model. Consistent bonded and nonbonded AMBER parameters for all TA atoms were assigned by analogy or through interpolation from those already present in the AMBER database^61^. Each of the individual building blocks were docked inside multiple boxes defined on the surface of the protein by using the Lamarckian genetic algorithm implemented in program AutoDock 4.2^62^. Protonation of the X-ray structure of the protein was achieved by means of the H++ server^63^ using a pH definition of 6.5 for the titratable side chains. Two RU of the TA were assembled by connecting the individual derivatized sugars from the most suitable docking poses with the appropriate glycosidic bond. In a second stage, restrained MD simulations in explicit solvent (TIP3P water molecules)^64^ were used to sample the conformational space of the TA wrapping around the positionally restrained protein and optimize the energy of the resulting complex. A standard protocol was followed using the *pmemd_cuda.SFSP* module from the AMBER14 suite of programs (http://ambermd.org/) that included positional restrains (5 kcal·mol^−1^·Å^−2^) on the nitrogen atoms of PCho. The remaining RU atoms were allowed to move in order to relax the whole TA molecule. Periodic boundary conditions were applied and electrostatic interactions were treated using the smooth particle mesh Ewald method with a grid spacing of 1 Å and a cutoff distance of 9 Å for the nonbonded interactions. The SHAKE algorithm was applied to all bonds and an integration step of 2.0 fs was used throughout. The molecular graphics program PyMOL version 1.8.1.0^60^ was employed for visualization and molecular editing.

### Generation of polyclonal anti-CbpL antisera

Antibodies against rCbpL were raised in 6 to 8 weeks old female CD-1 mice (Charles River Laboratories, Sulzfeld Germany) by immunizing mice intraperitoneally with 100 μL of a 1:1 emulsion containing 50 μg recombinant protein and incomplete Freund’s adjuvant (Sigma-Aldrich, Taufkirchen, Germany). Mice were boosted with an emulsion of protein and incomplete Freund´s adjuvant at day 14 and 28 and bled after six weeks.

### Phagocytosis experiments

Phagocytosis experiments were performed with wild-type and mutant pneumococci. Internalization and evaluation of intracellular survival of pneumococci in J774A.1 murine macrophages (DSMZ, Braunschweig, Germany) were conducted identical to the method described recently^38^,^43^,^65^.

Briefly, confluent monolayers of J774 cells in 24-well cell culture plates and cultured in RPMI1640/10% FBS (PAA Laboratories) were incubated for 30 min with pneumococci. Extracellular bacteria and non-adherent pneumococci were then killed by the antibiotic protection assay. The infected cells were washed three times with the infection medium and then replaced and incubated for 1 h with RPMI 1640 medium containing gentamicin (100 μg/mL) and penicillin G (100 units/mL) to kill non-internalized bacteria. Intracellular pneumococci were recovered by a saponin-mediated lysis (1% w/v) of macrophages^43^ and the number of recovered and viable pneumococci (survivors) was determined by quantitative plating of the released intracellular pneumococci on sheep blood agar plates. Experiments were conducted at least three times in triplicate. To investigate whether *cbpL* deletion has an influence on the intracellular survival of pneumococci the infected and antibiotics treated cells were either lysed immediately or one, two or three hours post antibiotics treatment. Experiments were conducted at least three times in duplicate. Fluorescence microscopy was performed to visualize attached or ingested pneumococci. Macrophages (10^5^) were seeded on glass cover slips (12 mm ∅) in wells of a 24-cell culture plate and two days later the cells were infected with pneumococci as described above. Post infection unbound bacteria were removed and the infected host cells were fixed on the glass cover slips with 3.7% paraformaldehyde) and double immunofluorescence microscopy was carried out as described^43^. Image acquisition was performed with a fluorescence microscope (Zeiss Axio-Observer.Z1 with VisiGrid, Coolsnap HQ and Visiview Imaging software (Visitron Systems GmbH, Puchheim, Germany). All experiments were performed at least three times with two or more replicate wells tested for each experimental setup.

### Mouse models of infection and bioluminescent optical imaging

Mouse infection experiments and real-time monitoring of intranasally infected mice was carried out identical to the recently described protocol^38^,^66^,^67^.

Briefly, eight weeks old female outbred CD1 mice (Charles River, Sulzfeld, Germany) were infected intranasally with pneumococci cultured to A_600_ = 0.35 in THY supplemented with 10% fetal bovine serum and the infection dose was adjusted to 1.0 × 10^7^ CFU in 25 μL for the intranasal route (n = 12). Before intranasal infection, mice were anesthetized by intraperitoneal injection of ketamine (Ketanest S; Pfizer Pharma, Karslruhe, Germany) and xylazine (Rompun^®^; Provet AG, Lyssach, Germany). Once anesthetized the animals were scuffed, with the nose held upright, and the bacterial suspension of 25 μL was administered intranasally by adding a series of small droplets into the nostrils for the mice to involuntarily inhale. The infection dose was confirmed by determination of the CFU after plating serial dilutions of the infection dose on blood agar plates. Bioluminescent optical imaging using the IVIS^**®**^ Spectrum Imaging System (Caliper Life Sciences, Hopkinton, U.S.) allowed monitoring of pneumococcal dissemination after intranasal infection^38^,^67^. At pre-chosen time intervals post-infection mice were imaged for 1 min to monitor dissemination of pneumococci into the lungs. A time series of the images was generated and the bioluminescent intensity (BLI) was determined by quantification of the total photon emission using the LivingImage^®^ 4.1 software package (Caliper Life Sciences).

### Flow cytometry and immunofluorescence microcopy

*S. pneumoniae* wild-type and isogenic *cbpL*-mutants were cultured in 30 mL THY to A_600_ = 0.35–0.4 and after sedimentation bacteria were washed with RPMI 1640/1% FBS (fetal bovine serum; PAA Laboratories, Colbe, Germany) and finally resuspended in 1 mL of RPMI 1640/1% FBS. To detect CbpL on the surface of pneumococci 2 × 10^8^ bacteria were incubated with mouse anti-CbpL polyclonal antisera (1:1000 dilution in PBS) or antisera of mice injected with PBS and incubated for 30 min at 4 °C. Samples were then washed twice with PBS/0.5% FBS and stained with secondary goat anti-mouse IgG coupled Alexa-Fluor-488 (Invitrogen). After 30 min incubation at 4 °C bacteria were washed twice with PBS/0.5% and then fixed with 2% formaldehyde. Flow cytometry was conducted with the FACSCalibur™ (BD Biosciences, Heidelberg, Germany) and the CellQuestPro Software 6.0. (BD Biosciences) was used for data acquisition while analysis of the data was performed with the software WinMDI 2.9. The bacteria were detected and gated as described previously^43^ and the forward scatter (FL1-H) in the histograms shows the increase in fluorescence intensity. To illustrate CbpL by fluorescence microscopy pneumococci were treated as described above. However, prior to fixation pneumococci were treated with anti-pneumococcal antibodies and secondary goat-anti-rabbit IgG Alexa coupled Alexa-Fluor-568 (Invitrogen). Fluorescence microscopy image acquisition was performed with a fluorescence microscope (Zeiss Axio-Observer.Z1 with VisiGrid, Coolsnap HQ and Visiview Imaging software (Visitron Systems GmbH, Puchheim, Germany). Contrast and brightness were adjusted with Adobe Photoshop CS5.

### Generation of subcellular protein fractions and choline chloride treatment

Pneumococci were cultured as described above. The bacteria were sedimented by centrifugation at 3270 × g for 10 minutes when the cultures reached OD_600_ of 0.35–0.4. The supernatant was collected and the bacterial pellet was divided in two different treatments. One fraction was resuspended in PBS with 5% choline chloride (ChCl) and incubated for 30 min at RT, while the second fraction one was remained untreated. Bacteria were then centrifuged at 3270 × g for 10 minutes and supernatants were transferred to a new 15 mL conical tube and treated with 10% TCA for protein precipitation o/n at 4 °C. The subcellular fractionation of pneumococcal proteins was performed as described earlier 38,68,69. Briefly, bacterial pellets were washed with PBS, centrifuged and incubated in 2,5 mL of lysis buffer 1 (100 mM tris-HCL, 20% Sucrose, 20 mM MgCl_2_, 5 mg/mL lyzozyme, 200 U/mL mutanolysin, and 1 mM PMSF, pH 8.0) and incubated for 30 min at 37 °C in a water bath. Following the incubation the samples were centrifuged at 3000 × g for 10 min at 4 °C, the supernatants were transferred to 15 mL conical tubes and treated with 10% TCA for protein precipitation o/n at 4 °C. The pellets were washed with PBS as above finally resuspended in 0.5 mL of sucrose buffer (20% Sucrose and 10 mM Tris-HCL, pH 8.0) and disrupted with 9.5 mL of disruption buffer (100 mM Tris-HCL, 1 mM EDTA and 1 mM PMSF). The mixture was then centrifuged at 4000 × g for 10 min at 4 °C to remove any undisrupted protoplasts. The supernatants were transferred to ultracentrifugation tubes and the cell fractionation was carried out by ultracentrifugation at 100.000 × g for 30 min at 4 °C. The supernatant fractions (cytoplasm) were transferred to a 15 mL conical tube and incubated with 10% TCA o/n at 4 °C for protein precipitation, while the pellets containing the membrane fractions were resuspended in PBS. All samples treated with TCA were processed as follows: The tubes were vigorously vortexed and centrifuged for 20 min at 20000 × g and 4 °C, the TCA-supernatant was discarded and the pellet was washed thoroughly with 100% cold acetone and centrifuged again at 20000 × g for 10 min at 4 °C. The acetone was discarded and the pellet was air dried for 5 to 10 minutes and finally resuspended in PBS. The protein concentrations of collected fractions were measured by Bradford assay and samples were stored at −20 °C until further analysis. Protein fractions were separated by SDS-PAGE and transferred on nitrocellulose by semi-dry blotting and immunoblot analysis carried out. Antibodies used were anti-CbpL antibody (1/1000 in PBS), anti-PspC (1/500 in PBS) and anti-enolase (1/10000 in PBS).

### Statistical Analysis

All data are reported as mean ± SD unless otherwise noted. Results were statistically analyzed using the unpaired two-tailed Student´s test. Kaplan-Meier survival curves were compared by the log rank test. *P*-values for bioluminescence measurements were calculated using the unpaired, one-tailed t-test for differences between groups, while differences of one group between days were analyzed by the paired t-test. Statistical significance was confirmed by ANOVA analysis with Bonferroni´s Multiple Comparison post-hoc test. A *P-*value < 0.05 was considered to be statistically significant.

### Ethics statement

All the animal experiments were conducted in strict accordance with the guidelines of the ethics committee at The University of Greifswald and the German regulations of the Society for Laboratory Animal Science (GVSOLAS) and the European Health Law of the Federation of Laboratory Animal Science Associations (FELASA). All experiments were approved by the Landesamt für Landwirtschaft, Lebensmittelsicherheit und Fischerei Mecklenburg – Vorpommern (LALLFV M-V, Rostock, Germany) and the LALLFV M-V ethical board (LALLF M-V permit no. 7221.3‐1.1‐006/09 and 7221.3‐1.1‐019/11). All efforts were made to minimize suffering and ensure the highest ethical standards.

## Additional Information

**Accession codes:** The atomic coordinates for the CbpL_CBM_ and the Excalibur domain of CbpL have been deposited in the Protein Data Bank under codes 4CNL and 5J8T respectively. The NMR assignments of the Excalibur domain have been also deposited in the BioMagResBank under accession number 30064.

**How to cite this article:** Gutiérrez-Fernández, J. *et al*. Modular Architecture and Unique Teichoic Acid Recognition Features of Choline-Binding Protein L (CbpL) Contributing to Pneumococcal Pathogenesis. *Sci. Rep.* **6**, 38094; doi: 10.1038/srep38094 (2016).

**Publisher's note:** Springer Nature remains neutral with regard to jurisdictional claims in published maps and institutional affiliations.

## Supplementary Material

Supplementary Information

## Figures and Tables

**Table 1:** Crystallographic data collection and refinement statistics for CbpL_CBM_.

Parameters	CbpL_CBM_
Data collection	
Space group	P2_1_
*a, b, c* (Å)	30.85, 42.79, 70.62
α, β, γ (°)	90, 101.06, 90
T (K)	100
Wavelength (Å)	0.97857
Resolution (Å)	42.8–1.7 (1.73–1.7)
No. unique reflections	19551
*R*_*pim*_ (%)^a^	1.8 (5.6)
CC* (%)	99.97 (99.85)
<*I*/σ(*I*)>	27.5 (11.1)
Completeness (%)	97.7 (96.8)
Multiplicity	6.7 (7.0)
Refinement	
Resolution (Å)	36.4–1.7
*R*_*work*_*/R*_*free*_^b^	0.15/0.19
*R. m. s. deviations*	
Bond length (Å)	0.006
Bond angles (°)	0.886
PDB code	4CNL

^a^*R*_pim_ = Σ_hkl_[1/(N − 1)] 1/2 Σ_i_ |I_i_(hkl) − [I(hkl)]|/Σ_hkl_Σ_i_I_i_(hkl), where Σ_i_I_i_(hkl) is the *i*-th measurement of reflection hkl, [I(hkl)] is the weighted mean of all measurements and N is the redundancy for the hkl reflection.

^b^*R*_work_/*R*_free_ = Σ_hkl_|F_o_ − F_c_|/Σ_hkl_ |F_o_|, where F_c_ is the calculated and F_o_ is the observed structure factor amplitude of reflection hkl for the working/free (5%) set, respectively.

**Table 2:** Structural statistics for the 20 best NMR structures of the Excalibur domain.

NOE distance restraints:	
Intraresidual	268
Medium range (within 5 residues)	118
Long range (5 or more residues)	178
Dihedral angle restraints	58
Hydrogen-bond restraints	0
Disulfide restraints	6
Metal Ion restrains	8
Stereospecific ^1^H assignments	12
Total (per residue)	648 (13.8)
Maximal violation (Å)	0.23
Number of violations >2.5 (Å)	0
CYANA target energy function (Å^2^)	1.15 ± 0.02
AMBER energy (kcal/mol)	−1974 ± 13
RMS deviations from ideal geometry:	
Bond Lengths (Å)	0.0112 ± 0.0001
Bond Angles (°)	2.52 ± 0.03
RMSD (to mean coordinates) 27–70:	
Backbone N, CA, C’(Å)	0.69
All heavy atoms (Å)	1.38
PROCHECK Ramachandran plot statistics:	
Most favored regions (%)	79.8
Additionally allowed favored regions (%)	19.9
Generously allowed regions (%)	0.3

Average values over the 20 final energy-minimized conformers. The CYANA target function value was computed for the structures before energy minimization with AMBER.

## References

[b1] Cartwright K. A. Epidemiology of meningococcal disease. Hosp Med 63, 264–267 (2002). 12066343 10.12968/hosp.2002.63.5.2017

[b2] Kadioglu A., Weiser J. N., Paton J. C. & Andrew P. W. The role of Streptococcus pneumoniae virulence factors in host respiratory colonization and disease. Nat Rev Microbiol 6, 288–301, doi: 10.1038/nrmicro1871 (2008). 18340341

[b3] Gamez G. & Hammerschmidt S. Combat Pneumococcal Infections: Adhesins as Candidates for Protein-Based Vaccine Development. Curr Drug Targets 13, 323–337 (2012). 22206255 10.2174/138945012799424697

[b4] Perez-Dorado I., Galan-Bartual S. & Hermoso J. A. Pneumococcal surface proteins: when the whole is greater than the sum of its parts. Mol Oral Microbiol 27, 221–245, doi: 10.1111/j.2041-1014.2012.00655.x (2012). 22759309

[b5] Mellroth P. . Structural and functional insights into peptidoglycan access for the lytic amidase LytA of Streptococcus pneumoniae. MBio 5, e01120–01113, doi: 10.1128/mBio.01120-13 (2014). 24520066 PMC3950521

[b6] Kietzman C. C., Gao G., Mann B., Myers L. & Tuomanen E. I. Dynamic capsule restructuring by the main pneumococcal autolysin LytA in response to the epithelium. Nat Commun 7, 10859, doi: 10.1038/ncomms10859 (2016). 26924467 PMC4773454

[b7] Hergott C. B. . Bacterial exploitation of phosphorylcholine mimicry suppresses inflammation to promote airway infection. J Clin Invest 125, 3878–3890, doi: 10.1172/JCI81888 (2015). 26426079 PMC4607119

[b8] Luo R. . Solution structure of choline binding protein A, the major adhesin of Streptococcus pneumoniae. EMBO J 24, 34–43, doi: 10.1038/sj.emboj.7600490 (2005). 15616594 PMC544903

[b9] Agarwal V. . Complement regulator Factor H mediates a two-step uptake of Streptococcus pneumoniae by human cells. J Biol Chem 285, 23486–23495, doi: 10.1074/jbc.M110.142703 (2010). 20504767 PMC2906339

[b10] Voss S. . The choline-binding protein PspC of Streptococcus pneumoniae interacts with the C-terminal heparin-binding domain of vitronectin. J Biol Chem 288, 15614–15627, doi: 10.1074/jbc.M112.443507 (2013). 23603906 PMC3668722

[b11] Binsker U. . Pneumococcal Adhesins PavB and PspC Are Important for the Interplay with Human Thrombospondin-1. J Biol Chem 290, 14542–14555, doi: 10.1074/jbc.M114.623876 (2015). 25897078 PMC4505522

[b12] Zhang J. R. . The polymeric immunoglobulin receptor translocates pneumococci across human nasopharyngeal epithelial cells. Cell 102, 827–837 (2000). 11030626 10.1016/s0092-8674(00)00071-4

[b13] Elm C. . Ectodomains 3 and 4 of human polymeric Immunoglobulin receptor (hpIgR) mediate invasion of Streptococcus pneumoniae into the epithelium. J Biol Chem 279, 6296–6304, doi: 10.1074/jbc.M310528200 (2004). 14660617

[b14] Claverys J. P. & Havarstein L. S. Cannibalism and fratricide: mechanisms and raisons d’etre. Nature Reviews Microbiology 5, 219–229, doi: 10.1038/nrmicro1613 (2007). 17277796

[b15] Perez-Dorado I. . Insights into pneumococcal fratricide from the crystal structures of the modular killing factor LytC. Nat Struct Mol Biol 17, 576–U572, doi: 10.1038/nsmb.1817 (2010). 20400948 PMC6902435

[b16] Molina R. . Crystal structure of CbpF, a bifunctional choline-binding protein and autolysis regulator from Streptococcus pneumoniae. EMBO Rep 10, 246–251, doi: 10.1038/embor.2008.245 (2009). 19165143 PMC2658566

[b17] Galán-Bartual S., Pérez-Dorado I., García P. & Hermoso J. In Streptococcus pneumoniae, molecular mechanisms of host-pathogen interactions (eds Brown J., Hammerschmidt S. & Orihuela C.) 207–230 (Elsevier, 2015).

[b18] Garcia J. L., Sanchez-Beato A. R., Medrano F. J. & Lopez R. Versatility of choline-binding domain. Microb Drug Resist 4, 25–36, doi: 10.1089/mdr.1998.4.25 (1998). 9533722

[b19] Rigden D. J., Jedrzejas M. J. & Galperin M. Y. An extracellular calcium-binding domain in bacteria with a distant relationship to EF-hands. Fems Microbiol Lett 221, 103–110, doi: 10.1016/S0378-1097(03)00160-5 (2003). 12694917

[b20] Bebeacua C. . X-ray structure of a superinfection exclusion lipoprotein from phage TP-J34 and identification of the tape measure protein as its target. Mol Microbiol 89, 152–165, doi: 10.1111/mmi.12267 (2013). 23692331

[b21] Chimalapati S. . Infection with Conditionally Virulent Streptococcus pneumoniae Delta pab Strains Induces Antibody to Conserved Protein Antigens but Does Not Protect against Systemic Infection with Heterologous Strains. Infect Immun 79, 4965–4976, doi: 10.1128/Iai.05923-11 (2011). 21947774 PMC3232651

[b22] Hava D. & Camilli A. Large-scale identificationof serotype 4 Streptococcus pneumoniae virulence factors. Mol Microbiol 45, 1389–1405, doi: 10.1046/j.1365-2958.2002.03106.x (2002). 12207705 PMC2788772

[b23] Novak R. . Extracellular targeting of choline-binding proteins in Streptococcus pneumoniae by a zinc metalloprotease. Mol Microbiol 36, 366–376, doi: 10.1046/j.1365-2958.2000.01854.x (2000). 10792723

[b24] Susskind M. M., Botstein D. & Wright A. Superinfection exclusion by P22 prophage in lysogens of Salmonella typhimurium. III. Failure of superinfecting phage DNA to enter sieA+ lysogens. Virology 62, 350–366 (1974). 4610992 10.1016/0042-6822(74)90398-5

[b25] Pribyl T. . Influence of impaired lipoprotein biogenesis on surface and exoproteome of Streptococcus pneumoniae. J Proteome Res 13, 650–667, doi: 10.1021/pr400768v (2014). 24387739

[b26] Hermoso J. A. . Insights into pneumococcal pathogenesis from the crystal structure of the modular teichoic acid phosphorylcholine esterase Pce. Nat Struct Mol Biol 12, 533–538, doi: 10.1038/nsmb940 (2005). 15895092

[b27] Holm L. & Rosenstrom P. Dali server: conservation mapping in 3D. Nucleic Acids Res 38, W545–549, doi: 10.1093/nar/gkq366 (2010). 20457744 PMC2896194

[b28] Gisch N. . Structural reevaluation of Streptococcus pneumoniae Lipoteichoic acid and new insights into its immunostimulatory potency. J Biol Chem 288, 15654–15667, doi: 10.1074/jbc.M112.446963 (2013). 23603911 PMC3668725

[b29] Frolet C. . New adhesin functions of surface-exposed pneumococcal proteins. Bmc Microbiol 10, doi: Artn 19010.1186/1471-2180-10-190 (2010). 10.1186/1471-2180-10-190 PMC2911433 20624274

[b30] Vollmer W. In Molecular biology of streptococci (eds Mitchell T. J., Morrison D. A. & Spratt B. G.) 83–118 (Norfolk: Horizon Bioscience, 2007).

[b31] Gisch N., Peters K., Zähringer U. & Vollmer W. In Streptococcus pneumoniae, molecular mechanisms of host-pathogen interactions (eds Brown J., Hammerschmidt S. & Orihuela C.) 145–168 (Elsevier, 2015).

[b32] Vollmer W. & Tomasz A. Identification of the teichoic acid phosphorylcholine esterase in Streptococcus pneumoniae. Mol Microbiol 39, 1610–1622 (2001). 11260477 10.1046/j.1365-2958.2001.02349.x

[b33] Rico-Lastres P. . Substrate recognition and catalysis by LytB, a pneumococcal peptidoglycan hydrolase involved in virulence. Sci Rep 5, 16198, doi: 10.1038/srep16198 (2015). 26537571 PMC4633669

[b34] Eldholm V. . Pneumococcal CbpD is a murein hydrolase that requires a dual cell envelope binding specificity to kill target cells during fratricide. Mol Microbiol 76, 905–917, doi: 10.1111/j.1365-2958.2010.07143.x (2010). 20384696

[b35] Ishida T. & Kinoshita K. PrDOS: prediction of disordered protein regions from amino acid sequence. Nucleic Acids Res 35, W460–464, doi: 10.1093/nar/gkm363 (2007). 17567614 PMC1933209

[b36] Standish A. J., Stroeher U. H. & Paton J. C. The pneumococcal two-component signal transduction system RR/HK06 regulates CbpA and PspA by two distinct mechanisms. J Bacteriol 189, 5591–5600, doi: 10.1128/JB.00335-07 (2007). 17526693 PMC1951833

[b37] Blue C. E. & Mitchell T. J. Contribution of a response regulator to the virulence of Streptococcus pneumoniae is strain dependent. Infect Immun 71, 4405–4413 (2003). 12874319 10.1128/IAI.71.8.4405-4413.2003 PMC166049

[b38] Saleh M. . Molecular architecture of Streptococcus pneumoniae surface thioredoxin-fold lipoproteins crucial for extracellular oxidative stress resistance and maintenance of virulence. EMBO Mol Med 5, 1852–1870, doi: 10.1002/emmm.201202435 (2013). 24136784 PMC3914529

[b39] Sivashanmugam A. . Practical protocols for production of very high yields of recombinant proteins using Escherichia coli. Protein Sci 18, 936–948, doi: 10.1002/pro.102 (2009). 19384993 PMC2771296

[b40] Hammerschmidt S., Talay S. R., Brandtzaeg P. & Chhatwal G. S. SpsA, a novel pneumococcal surface protein with specific binding to secretory immunoglobulin A and secretory component. Mol Microbiol 25, 1113–1124 (1997). 9350867 10.1046/j.1365-2958.1997.5391899.x

[b41] Schulz C. . Regulation of the arginine deiminase system by ArgR2 interferes with arginine metabolism and fitness of Streptococcus pneumoniae. MBio 5, doi: 10.1128/mBio.01858-14 (2014). PMC4278536 25538192

[b42] Rennemeier C. . Thrombospondin-1 promotes cellular adherence of gram-positive pathogens via recognition of peptidoglycan. FASEB J 21, 3118–3132, doi: 10.1096/fj.06-7992com (2007). 17507668

[b43] Jensch I. . PavB is a surface-exposed adhesin of Streptococcus pneumoniae contributing to nasopharyngeal colonization and airways infections. Mol Microbiol 77, 22–43, doi: 10.1111/j.1365-2958.2010.07189.x (2010). 20444103

[b44] Kabsch W. Xds. Acta Crystallogr D Biol Crystallogr 66, 125–132, doi: 10.1107/S0907444909047337 (2010). 20124692 PMC2815665

[b45] Evans P. R. & Murshudov G. N. How good are my data and what is the resolution? Acta Crystallogr D Biol Crystallogr 69, 1204–1214, doi: 10.1107/S0907444913000061 (2013). 23793146 PMC3689523

[b46] Matthews B. W. Solvent content of protein crystals. J Mol Biol 33, 491–497 (1968). 5700707 10.1016/0022-2836(68)90205-2

[b47] McCoy A. J. . Phaser crystallographic software. J Appl Crystallogr 40, 658–674, doi: 10.1107/S0021889807021206 (2007). 19461840 PMC2483472

[b48] Winn M. D. . Overview of the CCP4 suite and current developments. Acta Crystallogr D Biol Crystallogr 67, 235–242, doi: 10.1107/S0907444910045749 (2011). 21460441 PMC3069738

[b49] Adams P. D. . PHENIX: a comprehensive Python-based system for macromolecular structure solution. Acta Crystallogr D Biol Crystallogr 66, 213–221, doi: 10.1107/S0907444909052925 (2010). 20124702 PMC2815670

[b50] Emsley P., Lohkamp B., Scott W. G. & Cowtan K. Features and development of Coot. Acta Crystallogr D Biol Crystallogr 66, 486–501, doi: 10.1107/S0907444910007493 (2010). 20383002 PMC2852313

[b51] Chen V. B. . MolProbity: all-atom structure validation for macromolecular crystallography. Acta Crystallogr D Biol Crystallogr 66, 12–21, doi: 10.1107/S0907444909042073 (2010). 20057044 PMC2803126

[b52] Markley J. L. . Recommendations for the presentation of NMR structures of proteins and nucleic acids--IUPAC-IUBMB-IUPAB Inter-Union Task Group on the standardization of data bases of protein and nucleic acid structures determined by NMR spectroscopy. Eur J Biochem 256, 1–15 (1998). 9746340 10.1046/j.1432-1327.1998.2560001.x

[b53] Sun Z. Y., Frueh D. P., Selenko P., Hoch J. C. & Wagner G. Fast assignment of 15N-HSQC peaks using high-resolution 3D HNcocaNH experiments with non-uniform sampling. J Biomol NMR 33, 43–50, doi: 10.1007/s10858-005-1284-4 (2005). 16222556

[b54] Pantoja-Uceda D. & Santoro J. Aliasing in reduced dimensionality NMR spectra: (3,2)D HNHA and (4,2)D HN(COCA)NH experiments as examples. J Biomol NMR 45, 351–356, doi: 10.1007/s10858-009-9383-2 (2009). 19851713

[b55] Delaglio F. . NMRPipe: a multidimensional spectral processing system based on UNIX pipes. J Biomol NMR 6, 277–293 (1995). 8520220 10.1007/BF00197809

[b56] Goddard T. D. & Kneller D. G. SPARKY 3: NMR Assignment and Integration Software. University of California, San Francisco, USA. URL http://www.iop.vast.ac.vn/theor/conferences/smp/1st/kaminuma/UCSFComputerGraphicsLab/index-16.htm. (2002).

[b57] Johnson B. A. & Blevins R. A. NMR View: A computer program for the visualization and analysis of NMR data. J Biomol NMR 4, 603–614, doi: 10.1007/BF00404272 (1994). 22911360

[b58] Shen Y., Delaglio F., Cornilescu G. & Bax A. TALOS+: a hybrid method for predicting protein backbone torsion angles from NMR chemical shifts. J Biomol NMR 44, 213–223, doi: 10.1007/s10858-009-9333-z (2009). 19548092 PMC2726990

[b59] Koradi R., Billeter M. & Wuthrich K. MOLMOL: a program for display and analysis of macromolecular structures. J Mol Graph 14, 51–55, 29–32 (1996). 10.1016/0263-7855(96)00009-4 8744573

[b60] Team PyMOL The PyMOL Molecular Graphics System, Version 1.8 Schrödinger, LLC. URL https://www.pymol.org/ (2016).

[b61] Aytenfisu A. H., Spasic A., Seetin M. G., Serafini J. & Mathews D. H. Modified Amber Force Field Correctly Models the Conformational Preference for Tandem GA pairs in RNA. J Chem Theory Comput 10, 1292–1301, doi: 10.1021/ct400861g (2014). 24803859 PMC3985902

[b62] Morris G. M. . AutoDock4 and AutoDockTools4: Automated docking with selective receptor flexibility. J Comput Chem 30, 2785–2791, doi: 10.1002/jcc.21256 (2009). 19399780 PMC2760638

[b63] Anandakrishnan R., Aguilar B. & Onufriev A. V. H++ 3.0: automating pK prediction and the preparation of biomolecular structures for atomistic molecular modeling and simulations. Nucleic Acids Res 40, W537–541, doi: 10.1093/nar/gks375 (2012). 22570416 PMC3394296

[b64] Jorgensen W. L., Chandrasekhar J., Madura J. D., Impey R. & Klein M. Comparison of simple potential functions for simulating liquid water. J Chem Phys 79 (1983).

[b65] Härtel T. . Impact of glutamine transporters on pneumococcal fitness under infection-related conditions. Infect Immun 79, 44–58, doi: 10.1128/IAI.00855-10 (2011). 21078855 PMC3019899

[b66] Abdullah M. R. . Structure of the pneumococcal l,d-carboxypeptidase DacB and pathophysiological effects of disabled cell wall hydrolases DacA and DacB. Mol Microbiol 93, 1183–1206, doi: 10.1111/mmi.12729 (2014). 25060741

[b67] Saleh M. . Following in real time the impact of pneumococcal virulence factors in an acute mouse pneumonia model using bioluminescent bacteria. J Vis Exp, e51174, doi: 10.3791/51174 (2014). PMC4130511 24637643

[b68] Bergmann S., Rohde M., Chhatwal G. S. & Hammerschmidt S. alpha-Enolase of Streptococcus pneumoniae is a plasmin(ogen)-binding protein displayed on the bacterial cell surface. Mol Microbiol 40, 1273–1287 (2001). 11442827 10.1046/j.1365-2958.2001.02448.x

[b69] Andisi V. F. . Pneumococcal gene complex involved in resistance to extracellular oxidative stress. Infect Immun 80, 1037–1049, doi: 10.1128/IAI.05563-11 (2012). 22215735 PMC3294666

[b70] Noske N., Kammerer U., Rohde M. & Hammerschmidt S. Pneumococcal interaction with human dendritic cells: phagocytosis, survival, and induced adaptive immune response are manipulated by PavA. J Immunol 183, 1952–1963, doi: 10.4049/jimmunol.0804383 (2009). 19570831

[b71] Hammerschmidt S. . The host immune regulator factor H interacts via two contact sites with the PspC protein of Streptococcus pneumoniae and mediates adhesion to host epithelial cells. J Immunol 178, 5848–5858 (2007). 17442969 10.4049/jimmunol.178.9.5848

[b72] Hermans P. W. . The streptococcal lipoprotein rotamase A (SlrA) is a functional peptidyl-prolyl isomerase involved in pneumococcal colonization. J Biol Chem 281, 968–976, doi: 10.1074/jbc.M510014200 (2006). 16260779

