# Supplementary material for: Modular Architecture and Unique Teichoic Acid Recognition Features of Choline-Binding Protein L (CbpL) Contributing to Pneumococcal Pathogenesis
Source: Sci Rep. 2016 Dec 5;6:38094. doi: 10.1038/srep38094 (PMC5137146; doi:10.1038/srep38094)
Supplement: Supplementary Information [file srep38094-s1.pdf]

SUPPORTING INFORMATION

Modular Architecture and Unique Teichoic Acid Recognition Features of Choline-Binding Protein L (CbpL) Contributing to Pneumococcal Pathogenesis

Javier Gutiérrez-Fernández,[1,5,6] Malek Saleh,[2,5,6] Martín Alcorlo,[1,5] Alejandro Gómez M.,[2] David Pantoja-Uceda,[3] Miguel A. Treviño,[3] Franziska Voß,[2] Mohammed R. Abdullah,[2] Sergio Galán-Bartual,[1,6] Jolien Seinen,[2] Pedro A. Sánchez-Murcia,[4] Federico Gago,[4] Marta Bruix,[3] Sven Hammerschmidt[2*] and Juan A. Hermoso[1*]

(1) Department of Crystallography and Structural Biology, "Rocasolano" Institute of Physical-Chemistry, CSIC, Serrano 119, E-28006-Madrid, Spain.

(2) Department Genetics of Microorganisms, Interfaculty Institute for Genetics and Functional Genomics, Ernst Moritz Arndt University of Greifswald, D-17487 Greifswald, Germany.

(3) Department of Biological Physical Chemistry. "Rocasolano" Institute of Physical-Chemistry, CSIC, Serrano 119, E-28006-Madrid, Spain.

(4) Department of Biomedical Sciences, Unidad Asociada al IQM-CSIC, Universidad de Alcalá, E-28871 Alcalá de Henares, Madrid, Spain.

(5) These authors contributed equally to this work.

(6) Present address: J G-F: Institute of Science and Technology Austria (IST Austria), Am Campus 1, 3400 Klosterneuburg, Austria; MS: Institut für Biologie – Mikrobiologie, Freie Universität Berlin, 14195 Berlin, Germany; S G-B: School of Life Sciences, University of Dundee, Dundee, UK.

```
        10         20         30         40         50         60
MNKRLFSKMS LVTLPILALF SQSVLAEENI HFSSCKEAWA NGYSDIHEGE PGYSAKLDRD

        70         80         90        100        110        120
HDGVACELKN APKGAFKAKQ STAIQINTSS ATTSGWVKQD GAWYYFDGNG NLVKNAWQGS
                                                 R1
       130        140        150        160        170        180
YYLKADGKMA QSEWIYDSSY QAWYYLKSDG SYAKNAWQGA YYLKSNGKMA QGEWVYDSSY
   R2                    R3                   R4
       190        200        210        220        230        240
QAWYYLKSDG SYARNAWQGN YYLKSDGKMA KGEWVYDATY QAWYYLTSDG SYAYSTWQGN
   R5                    R6                     R7
       250        260        270        280        290        300
YYLKSDGKMA VNEWVDGGRY YVGADGVWKE VQASTASSSN DSNSEYSAAL GKAKSYNSLF
    R8                  R9
       310        320        330
HMSKKRMYRQ LTSDFDKFSN DAAQYAIDHL DD
```

Fig. S1. Amino acid sequence of CbpL.

Color code for the different parts of the protein is as follows: signal peptide (gray), Excalibur domain (green), linkers (black), choline binding domain (CBD, purple), Ltp_Lipoprotein domain (red). Sequence of the choline binding repeats (R1 to R9) integrating the CBD is underlined in different colors.

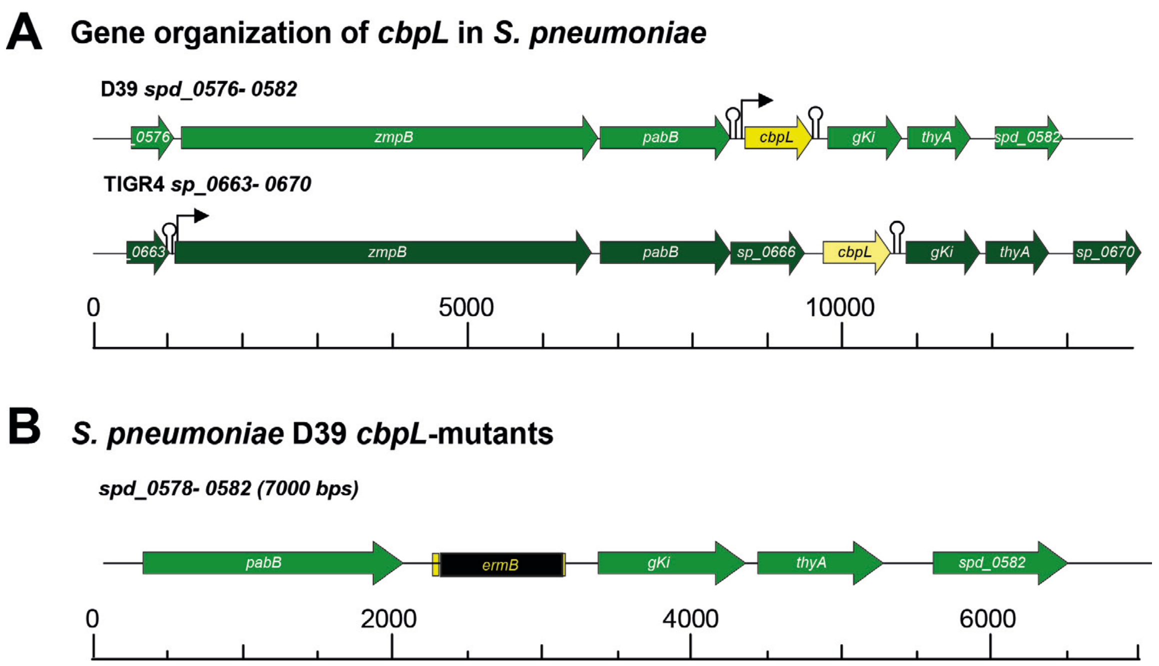

Fig. S2. Molecular organization of the *cbpL*-gene loci in *Streptococcus pneumoniae* D39 and TIGR4.

(**A**) The *in silico* analysis revealed a monocistronic organization of the CbpL-encoding gene in D39 and a polycistronic organization in TIGR4. The protein accession is YP_816078.1. The two genes upstream of *cbpL* in D39 encode a zinc metalloprotease (ZmpB; SPD_0577 in D39) and a *para*-aminobenzoic acid synthetase (PabB; SPD_0578 in D39). The genes downstream of *cbpL* encode a glucokinase (Gki; SPD_0580) and thymidylate synthase (ThyA; SPD_0581), respectively. In TIGR4 the gene *sp_0666* is annotated between *cbpL* and *pabB* and suggested to encode a putative pyrimidine utilization protein [1]. Putative promoters (arrows) were predicted by the Neural Network Promoter Prediction program (http://www.fruitfly.org/seq_tools/promoter.html) and potential rho-independent termination sequences were extracted from the TransTermHPTerminator Prediction list of *S. pneumoniae* D39 (http://transterm.cbcb.umd.edu/tt/Streptococcus_pneumoniae_D39.tt). The sequences

were obtained from the Kyoto Encyclopedia for Genes and Genomes (KEGG). (**B**) Mutants deficient in CbpL-expression were constructed by deletion-insertion mutagenesis. This resulted in a complete deletion of the *cbpL* gene sequence and insertion of an *ermB* gene cassette.

Pneumococcal CbpL protein sequence alignment

```
D39             SPD_0579          MNKRLFLKMS LVTLPILALF SQPVLAEENI HFSSCKEAWA NGYSDIHEGE PGYSAKLDRD   60 aa
R6              SPR_0583          MNKRLFLKMS LVTLPILALF SQPVLAEENI HFSSCKEAWA NGYSDIHEGE PGYSAKLDRD
TIGR4           SP_0667           MNKRLFSKMS LVTLPILALF SQSVLAEENI HFSSCKEAWA NGYSDIHEGE PGYSAKLDRD
OXC141          SPNOXC_06120      MNKRLFLKMS LVTLPILALF SQPVLAEENI HFSSCKEAWA NGYSDIYEGE PGYSAKLDRD
SPN034156       SPN034156_16610   MNKRLFLKMS LVTLPILALF SQPVLAEENI HFSSCKEAWA NGYSDIYEGE PGYSAKLDRD
AP200           SPAP_0656         MNKRLFLKMS LVTLPILALF SQPVLAEENI HFSSCKEAWA NGYSDLHEGE PGYSAKLDRD
CGSP14          SPCG_0623         MNKRLFLKMS LVTLPILALF SQPVLAEENI HFSSCKEAWA NGYSDIHEGE PGYSAKLDRD
INV200          SPNINV200_05890   MNKRLFLKMS LVTLPILALF SQPVLAEENI HFSSCKEAWA NGYSDIHEGE PGYSAKLDRD
Taiwan19f-14    SPT_0691          MNKRLFLKMS LVTLPILALF SQPVLAEENI HFSSCKEAWA NGYSDIHEGE PGYSAKLDRD
Hungary19A6     SPH_0762          MNKRLFLKMS LVTLPILALF SQPVLAEENI HFSSCKEAWA NGYSDIHEGE PGYSAKLDRD
G54             SPG_0608          MNKRLFLKMS LVTLPILALF SQPVLAEENI HFSSCKEAWA NGYSDIHEGE PGYSAKLDRD
SPN994039       SPN994039_06030   MNKRLFLKMS LVTLPILALF SQPVLAEENI HFSSCKEAWA NGYSDIYEGE PGYSAKLDRD
                                  ****** *** ********** **.******* ********** *****::*** **********

D39             SPD_0579          HDGVACELKN APKGAFKAKQ STAIQINTSS ATTSGWVKQD GAWYYFDGNG NLVKNAWQGS   120 aa
R6              SPR_0583          HDGVACELKN APKGAFKAKQ STAIQINTSS ATTSGWVKQD GAWYYFDGNG NLVKNAWQGS
TIGR4           SP_0667           HDGVACELKN APKGAFKAKQ STAIQINTSS ATTSGWVKQD GAWYYFDGNG NLVKNAWQGS
OXC141          SPNOXC_06120      HDGVACELKN APKGAFKAKQ STAIQINTSS ATTSGWVKQD GAWYYFDGNG NLVKNAWQGS
SPN034156       SPN034156_16610   HDGVACELKN APKGAFKAKQ STAIQINTSS ATTSGWVKQD GAWYYFDGNG NLVKNAWQGS
AP200           SPAP_0656         HDGVACELKN APKGAFKAKQ STAIQINTSS ATTSGWVKQD GAWYYFDGNG NLVKNAWQGS
CGSP14          SPCG_0623         HDGVACELKN APKGAFKAKQ STAIQINTSS ATTSGWVKQD GAWYYFDGNG NLVKNAWQGS
INV200          SPNINV200_05890   HDGVACELKN APKGAFKAKQ STAIQINTSS ATTSGWVKQD GAWYYFDGNG NLVKNAWQGS
Taiwan19f-14    SPT_0691          HDGVACELKN APKGAFKAKQ AATTQTDTTS STASGWVKQD GSWYYFDGNG NLVKNAWQGN
Hungary19A6     SPH_0762          HDGVACELKN APKGAFKAKQ STAIQINTSS ATTSGWVKQD GAWYYFDGNG NLVKNAWQGS
G54             SPG_0608          HDGVACELKN APKGAFKAKQ STAIQINTSS ATTSGWVKQD GAWYYFDGNG NLVKNAWQGS
SPN994039       SPN994039_06030   HDGVACELKN APKGAFKAKQ STAIQINTSS ATTSGWVKQD GAWYYFDGNG NLVKNAWQGS
                                  ********** ********** ::: * :*:* :*:******* *:******** *********.

D39             SPD_0579          YYLKADGKMA QSEWIYDSSY QAWYYLKSDG SYAKNAWQGA YYLKSNGKMA QGEWVYDSSY   180 aa
R6              SPR_0583          YYLKADGKMA QSEWIYDSSY QAWYYLKSDG SYAKNAWQGA YYLKSNGKMA QGEWVYDSSY
TIGR4           SP_0667           YYLKADGKMA QSEWIYDSSY QAWYYLKSDG SYAKNAWQGA YYLKSNGKMA QGEWVYDSSY
OXC141          SPNOXC_06120      YYLKADGKMA QSEWIYDSSY QAWYYLKSDG SYAKNAWQGA YYLKSNGKMV QGEWVYDSSY
SPN034156       SPN034156_16610   YYLKADGKMA QSEWIYDSSY QAWYYLKSDG SYAKNAWQGA YYLKSNGKMV QGEWVYDSSY
AP200           SPAP_0656         YYLKADGKMA QSEWIYDSSY QAWYYLKSDG SYAKNAWQGA YYLKSNGKMA QGEWVYDSSY
CGSP14          SPCG_0623         YYLKADGKMA QSEWIYDSSY QAWYYLKSDG SYAKNAWQGA YYLKSNGKMA QGEWVYDSSY
INV200          SPNINV200_05890   YYLKADGKMA QSEWIYDSSY QAWYYLKSDG SYAKNAWKGA YYLKSNGKMA QGEWVYDSSY
Taiwan19f-14    SPT_0691          YYLKADGKMA QSEWIYDSSY QAWYYLKSDG SYARNAWQGN YYLKSDGKMA KGEWVYDATY
Hungary19A6     SPH_0762          YYLKADGKMA QSEWIYDSSY QAWYYLKSDG SYAKNAWQGA YYLKSNGKMA QGEWVYDSSY
G54             SPG_0608          YYLKADGKMA QSEWIYDSSY QAWYYLKSDG SYAKNAWQGA YYLKSNGKMA QGEWVYDSSY
SPN994039       SPN994039_06030   YYLKADGKMA QSEWIYDSSY QAWYYLKSDG SYAKNAWQGA YYLKSNGKMV QGEWVYDSSY
                                  ********** ********** ********** ***:***:*  *****:***. :******::*

D39             SPD_0579          QAWYYLKSDG SYARNAWQGN YYLKSDGKMA KGEWVYDATY QAWYYLTSDG SYAYSTWQGN   240 aa
R6              SPR_0583          QAWYYLKSDG SYARNAWQGN YYLKSDGKMA KGEWVYDATY QAWYYLTSDG SYAYSTWQGN
TIGR4           SP_0667           QAWYYLKSDG SYARNAWQGN YYLKSDGKMA KGEWVYDATY QAWYYLTSDG SYAYSTWQGN
OXC141          SPNOXC_06120      QAWYYLKSDG ---------- -------KMA VNEWVYDATY QAWYYLTSDG SYAYSTWQGN
SPN034156       SPN034156_16610   QAWYYLKSDG ---------- ---------- ---------- ---------- SYARNAWQGN
AP200           SPAP_0656         QAWYYLKSDG SYARNAWQGN YYLKSDGKMA KGEWVYDATY QAWYYLTSDG SYAYSTWQGN
CGSP14          SPCG_0623         QAWYYLKSDG SYARNAWQGN YYLKSDGKMA KGEWVYDATY QAWYYLTSDG SYAYSTWQGN
INV200          SPNINV200_05890   QAWYYLKSDG SYARNAWQGN YYLKSDGKMA KGEWVYDATY QAWYYLTSDG SYAYSTWQGN
Taiwan19f-14    SPT_0691          QAWYYLKSDG SYARNAWQGN YYLKSDGKMA KGEWVYDATY QAWYYLTSDG SYAYSTWQGN
Hungary19A6     SPH_0762          QAWYYLKSDG SYARNAWQGN YYLKSDGKMA KGEWVYDATY QAWYYLTSDG SYAYSTWQGN
G54             SPG_0608          QAWYYLKSDG SYARNAWQGN YYLKSDGKMA KGEWVYVATY QAWYYLTSDG SYAYSTWQGN
SPN994039       SPN994039_06030   QAWYYLKSDG SYARNAWQGN YYLKSDGKMA VNEWVYDATY QAWYYLTSDG SYAYSTWQGN
                                  **********                                             *** .:****

D39             SPD_0579          YYLKSDGKMA VNEWVDGGRY YVGADGVWKE GQASTASSSN DSNSEYSAAL GKAKSYNSLF   300 aa
R6              SPR_0583          YYLKSDGKMA VNEWVDGGRY YVGADGVWKE GQASTASSSN DSNSEYSAAL GKAKSYNSLF
TIGR4           SP_0667           YYLKSDGKMA VNEWVDGGRY YVGADGVWKE VQASTASSSN DSNSEYSAAL GKAKSYNSLF
OXC141          SPNOXC_06120      YYLKSDGKMA VNEWVDGGRY YVGADGVWKE GQASTASSSN DSNSEYSAAL GKAKSYNSLF
SPN034156       SPN034156_16610   YYLKSDGKMA VNEWVDGGRY YVGADGVWKE GQASTASSSN DSNSEYSAAL GKAKSYNSLF
AP200           SPAP_0656         YYLKSDGKMA VNEWVDGGRY YVGADGVWKE GQASTASSSN DSNSEYSAAL GKAKSYNSLF
CGSP14          SPCG_0623         YYLKSDGKMA VNEWVDGGRY YVGADGVWKE GQASTVSSSN DSNSEYSAAL GKAKSYNSLF
INV200          SPNINV200_05890   YYLKSDGKMA VNEWVDGGRY YVGADGVWKE GQASTVSSSN DSNSEYSAAL GKAKSYNSLF
Taiwan19f-14    SPT_0691          YYLKSDGKMA VNEWVDGGRY YVGADGVWKE GQASTASSSN DSNSEYSAAL GKAKTYNSLF
Hungary19A6     SPH_0762          YYLKSDGKMA VNEWVDGGRY YVGADGVWKE GQASTASSSN DSNSEYSAAL GKAKSYNSLF
G54             SPG_0608          YYLKSDGKMA VNEWVDGGRY YVGADGVWKE GQASTASSSN DSNSEYSAAL GKAKSYNSLF
SPN994039       SPN994039_06030   YYLKSDGKMA VNEWVDGGRY YVGADGVWKE GQASTASSSN DSNSEYSAAL GKAKSYNSLF
                                  ********** ********** **********  ****.**** ********** ****:*****

D39             SPD_0579          HMSKKRMYRQ LTSDFDKFSN DAAQYAIDH- --   332 aa
R6              SPR_0583          HMSKKRMYRQ LTSDFDKFSN DAAQYAIDH- --
TIGR4           SP_0667           HMSKKRMYRQ LTSDFDKFSN DAAQYAIDHL DD
OXC141          SPNOXC_06120      HMSKKTYV-- ---------- ---------- --
SPN034156       SPN034156_16610   HMSKKTYV-- ---------- ---------- --
AP200           SPAP_0656         HMSKKRMYRQ LTSDFDKFSN DAAQYAIDHL DD
CGSP14          SPCG_0623         HMSKKRMYRQ LTSDFDKFSN DAAQYAIDHL DD
INV200          SPNINV200_05890   HMSKKRMYRQ LTSDFDKFSN DAAQYAIDHL DD
Taiwan19f-14    SPT_0691          HMSKKRMYRQ LTSDFDKFSN DAAQYAIDHL DD
Hungary19A6     SPH_0762          HMSKKTYV-- ---------- ---------- --
G54             SPG_0608          HMSKKTYV-- ---------- ---------- --
SPN994039       SPN994039_06030   HMSKKTYV-- ---------- ---------- --
                                  *****
```

signal peptide
Excalibur domain (DxDXDGXXCE)
Choline Binding Module (CBM)
Ltp-Lipoprotein domain

Fig. S3. Multiple amino acid sequence alignment of pneumococcal CbpL.

Alignment performed with Clustal Omega alignment tool (http://www.ebi.ac.uk/Tools/msa/clustalo/) with *cbpL* sequences as deposited in databases for 12 strains.

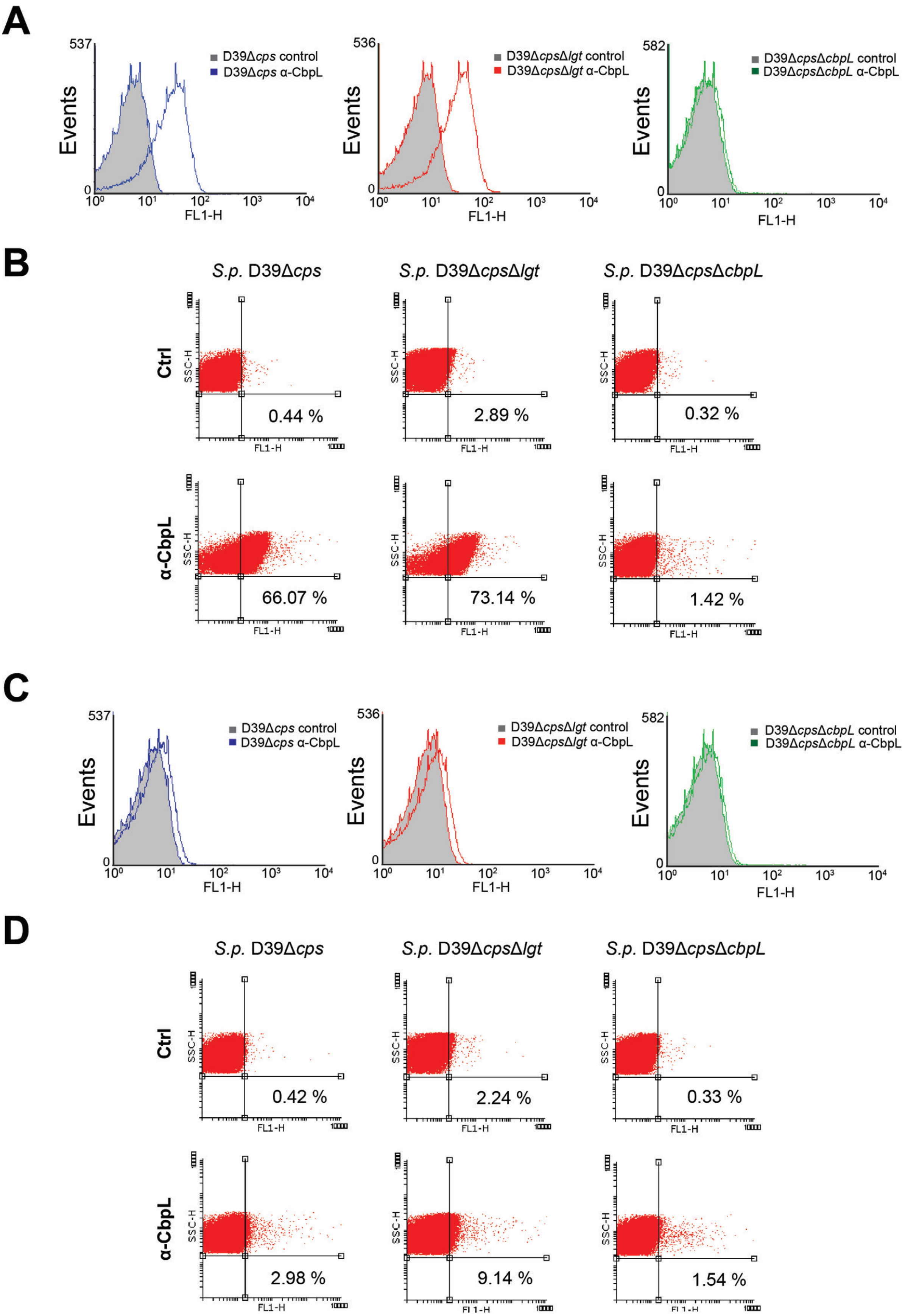
A
D39Δcps control
D39Δcps α-CbpL
D39ΔcpsΔlgt control
D39ΔcpsΔlgt α-CbpL
D39ΔcpsΔcbpL control
D39ΔcpsΔcbpL α-CbpL
Events
FL1-H
537
536
582
B
S.p. D39Δcps
S.p. D39ΔcpsΔlgt
S.p. D39ΔcpsΔcbpL
Ctrl
α-CbpL
SSC-H
FL1-H
0.44 %
2.89 %
0.32 %
66.07 %
73.14 %
1.42 %
C
D39Δcps control
D39Δcps α-CbpL
D39ΔcpsΔlgt control
D39ΔcpsΔlgt α-CbpL
D39ΔcpsΔcbpL control
D39ΔcpsΔcbpL α-CbpL
Events
FL1-H
537
536
582
D
S.p. D39Δcps
S.p. D39ΔcpsΔlgt
S.p. D39ΔcpsΔcbpL
Ctrl
α-CbpL
SSC-H
FL1-H
0.42 %
2.24 %
0.33 %
2.98 %
9.14 %
1.54 %

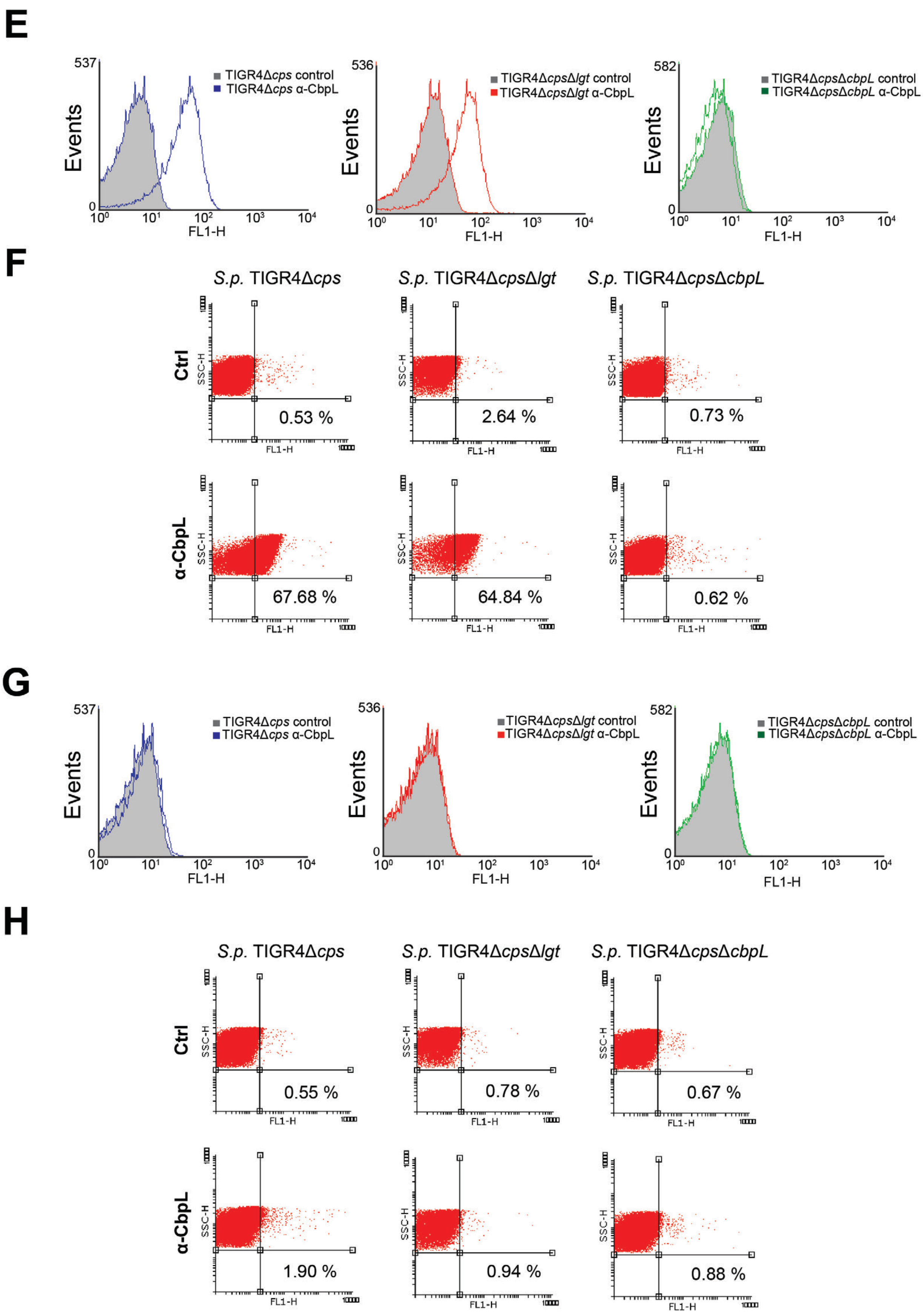

Fig. S4. Surface abundance of CbpL determined by flow cytometry.

The surface abundance of CbpL was measured by flow cytometry using anti-CbpL polyclonal antibodies (1:500) as primary antibodies and secondary goat anti-mouse IgG coupled Alexa-Fluor-488 (1:1000; Invitrogen). The non-encapsulated D39Δ*cps* (**A – D**) or TIGR4Δ*cps* (**E – H**) and their isogenic *lgt*- and *cbpL* mutants were incubated with the antibodies and the fluorescence intensity measured in a FACSCalibur™. The surface abundance of CbpL was measured prior (**A – B** and **E – F**) and post-treatment (**C – D** and **G – H**) of pneumococci with choline chloride (10% ChCl for 30 min). The individual histograms showing the increase in fluorescence intensity (forward scatter: FL1-H) for each strain tested (control: only the secondary anti-mouse IgG Alexa-Fluor conjugate antibody; CbpL detection: incubation with 1st and 2nd antibodies) are shown in **A, C, E**, and **G**. Dot plots of the flow cytometric analyses including the percent of positive events when counting 50000 events are shown in **B, D, F**, and **H**.

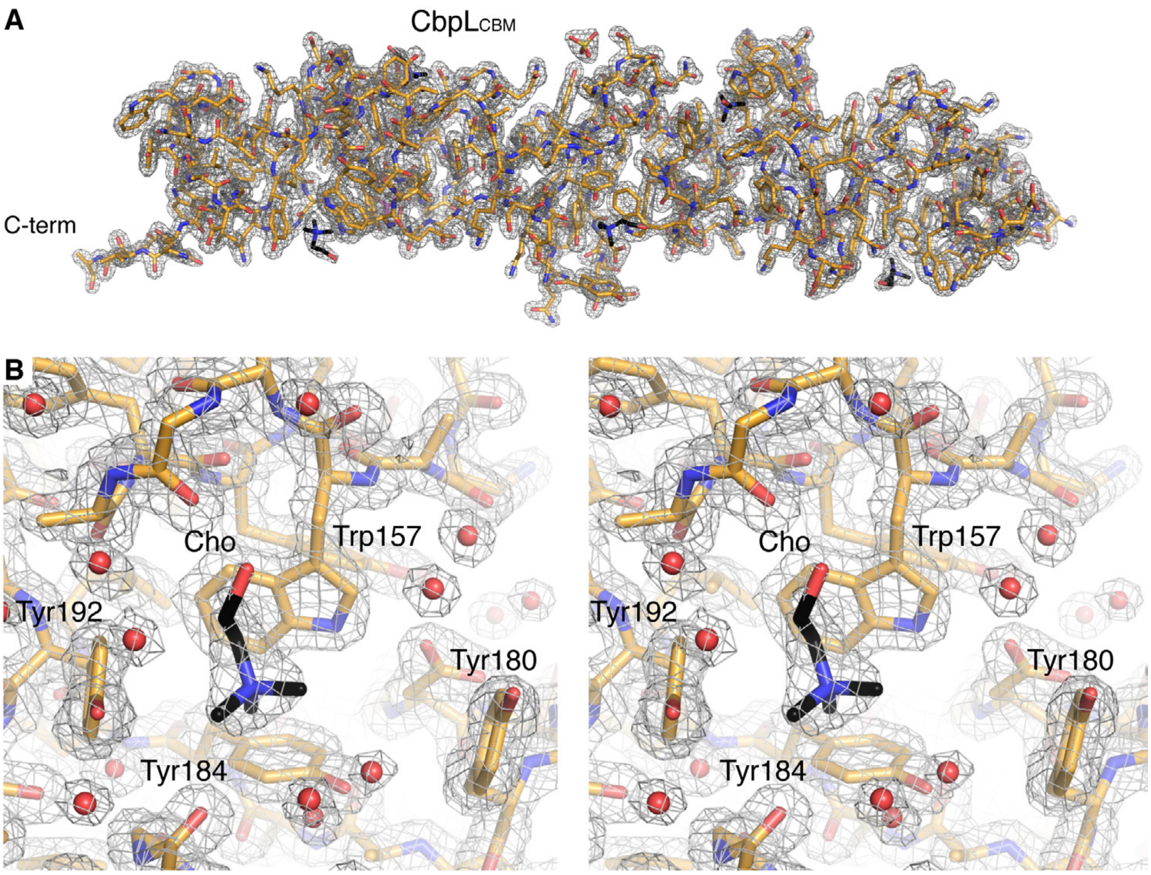

Fig. S5. Electron density in CbpL$_{CBM}$.

(**A**) Electron density map for the complete CbpL$_{CBM}$:choline complex. (**B**) Stereo view showing a detail of the electron density map in a non-canonical choline-binding site. Residues interacting with choline are represented as capped sticks and labeled. Water molecules represented as red spheres. Electron density maps correspond to the σ-weighted 2Fo–Fc electron density map contoured at 1.0 σ.

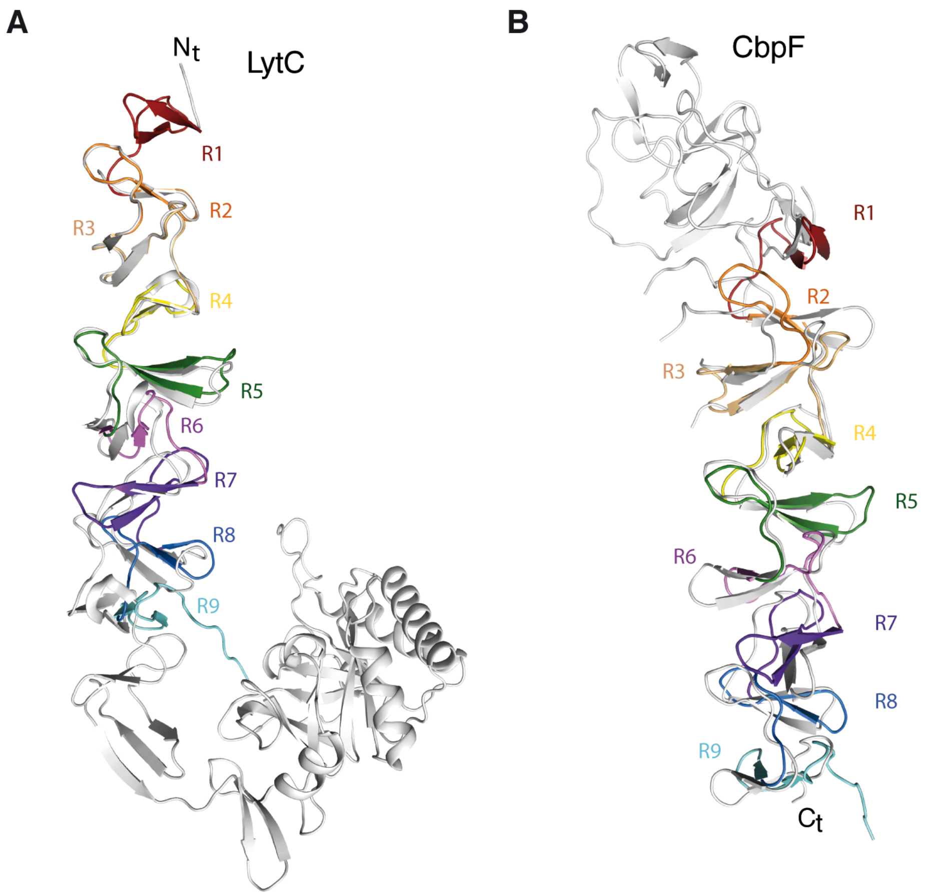

Fig. S6. Structural comparison of CbpL$_{CBM}$ with closest structural homologues.

(**A**) Structural superimposition of pneumococcal autolysin LytC (colored in grey) (PDB code 2WWD) with CbpL$_{CBM}$ (each repeat colored differently) results in a rmsd of 1.9 Å for 155 Cα atoms. (**B**) Structural superimposition of pneumococcal CbpF (colored in grey) (PDB code 2V05) with CbpL$_{CBM}$ (each repeat colored differently) results in a rmsd of 3.1 Å for 173 Cα atoms.

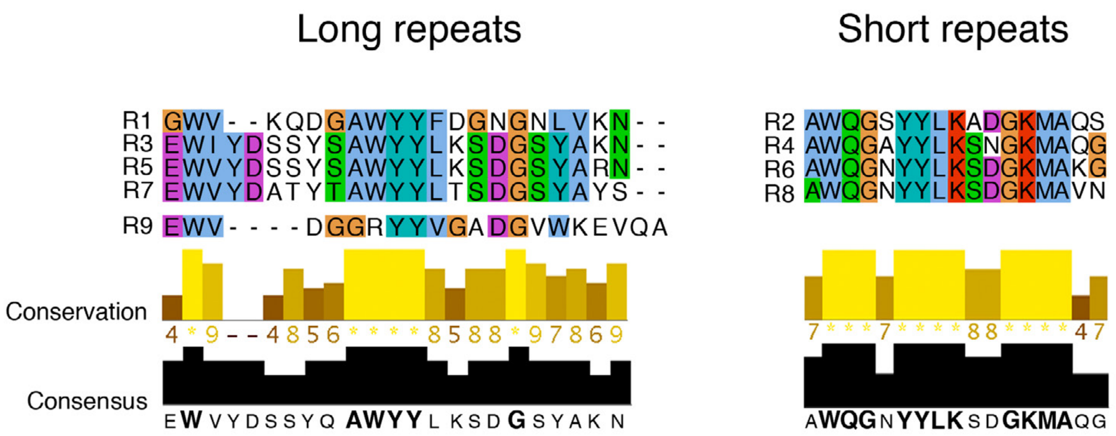

Fig. S7. Sequence comparison of choline-binding repeats in CbpL.

Long choline-binding repeats (left) and short choline-binding repeats (right) in CbpL are compared. Sequence alignments were performed with Clustal Omega and visualized using Jalview. R9 was not considered for the alignments.

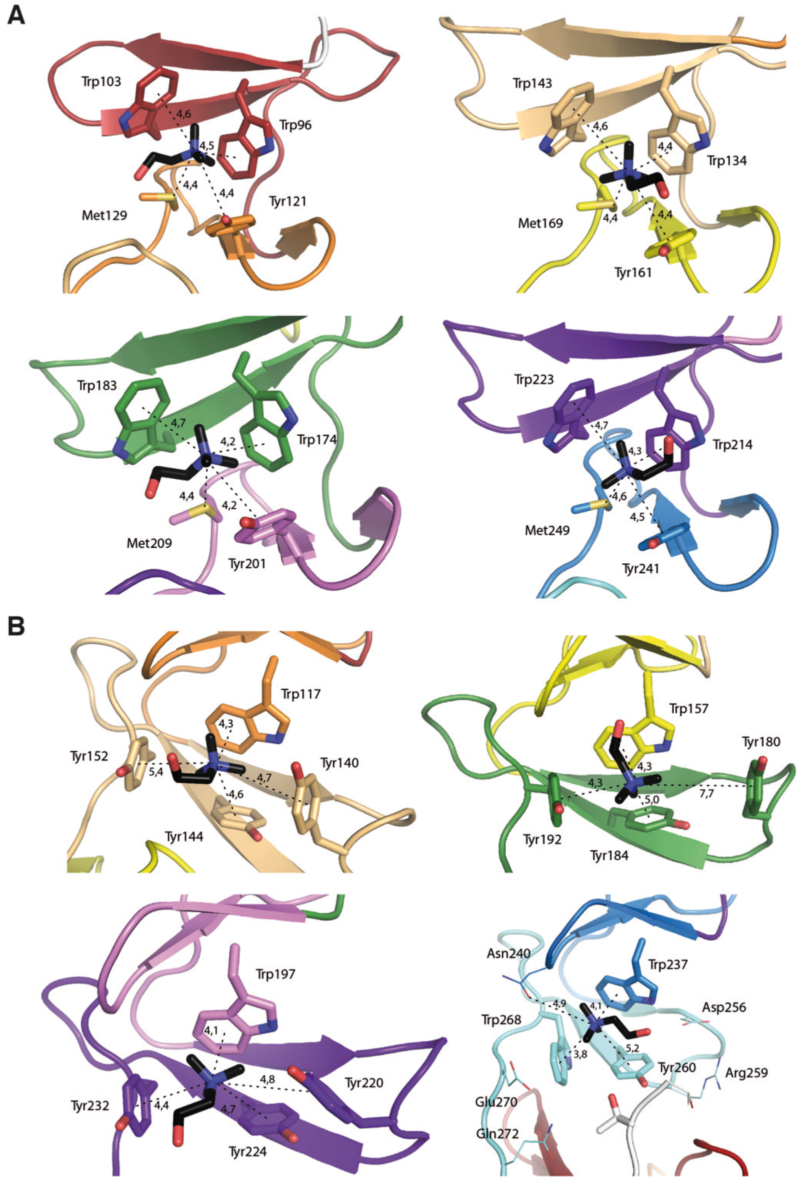

Fig. S8. Structural description of choline-binding sites in CbpL.

(**A**) The four canonical choline-binding sites present in CbpL (repeats are colored as in Fig 3). (**B**) The four non-canonical choline-binding sites present in CbpL (colored as in Fig 3) with main residues involved in choline stabilization depicted as capped sticks. Choline moieties attached to the choline-binding sites are represented as capped sticks with C atoms in black.

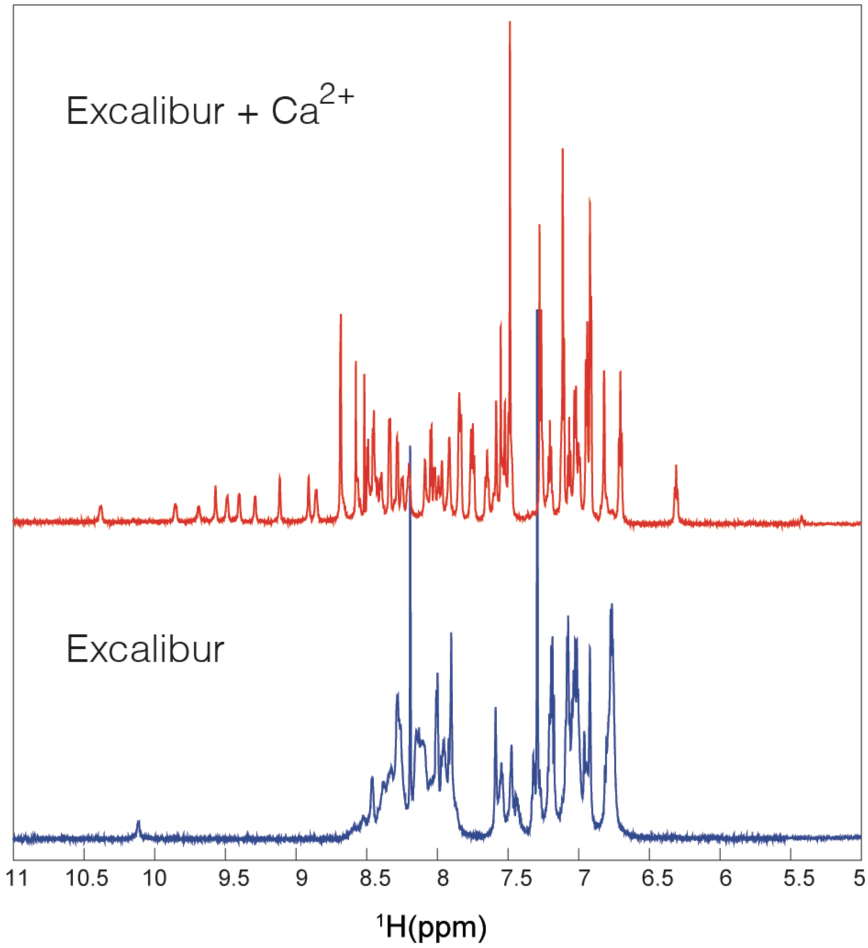

Fig. S9. Folding of the Excalibur domain requires Ca^{2+} binding.

NH region of the ^{1}H NMR spectra of Excalibur, 0.5 mM, pH 5.5, 800 MHz. Without Ca^{2+} the spectrum (colored in blue) shows severe signal overlapping in a narrow chemical shift range compatible with an unfolded peptide conformation. The same spectrum saturated with Ca^{2+} (colored in red) shows a large signal dispersion characteristic of a folded structure.

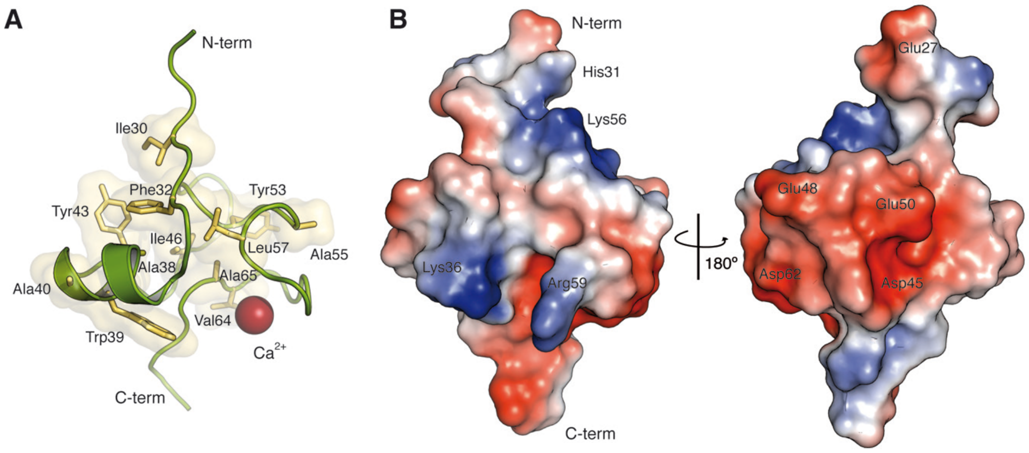

Fig. S10. Structural features of the Excalibur domain.

(**A**) A strong hydrophobic core is built with aromatic and hydrophobic residues, some of them are exposed. (**B**) Electrostatic potential surface of the Excalibur domain presents two faces, one of them with prevalence of positively-charged residues and the other (roughly 180º apart) presenting negatively-charged residues.

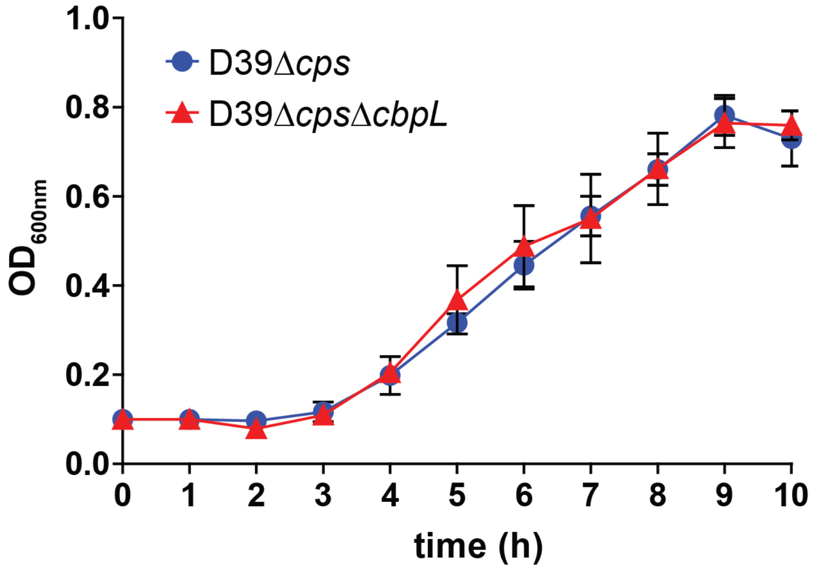

Fig. S11. Growth of pneumococci is not affected in pneumococci lacking CbpL. Pneumococci were cultured in the chemically defined medium $RPMI_{modi}$ [2] under microaerophilic conditions at 37 °C. At each indicated time point the optical density of the culture was measured at 600 nm.

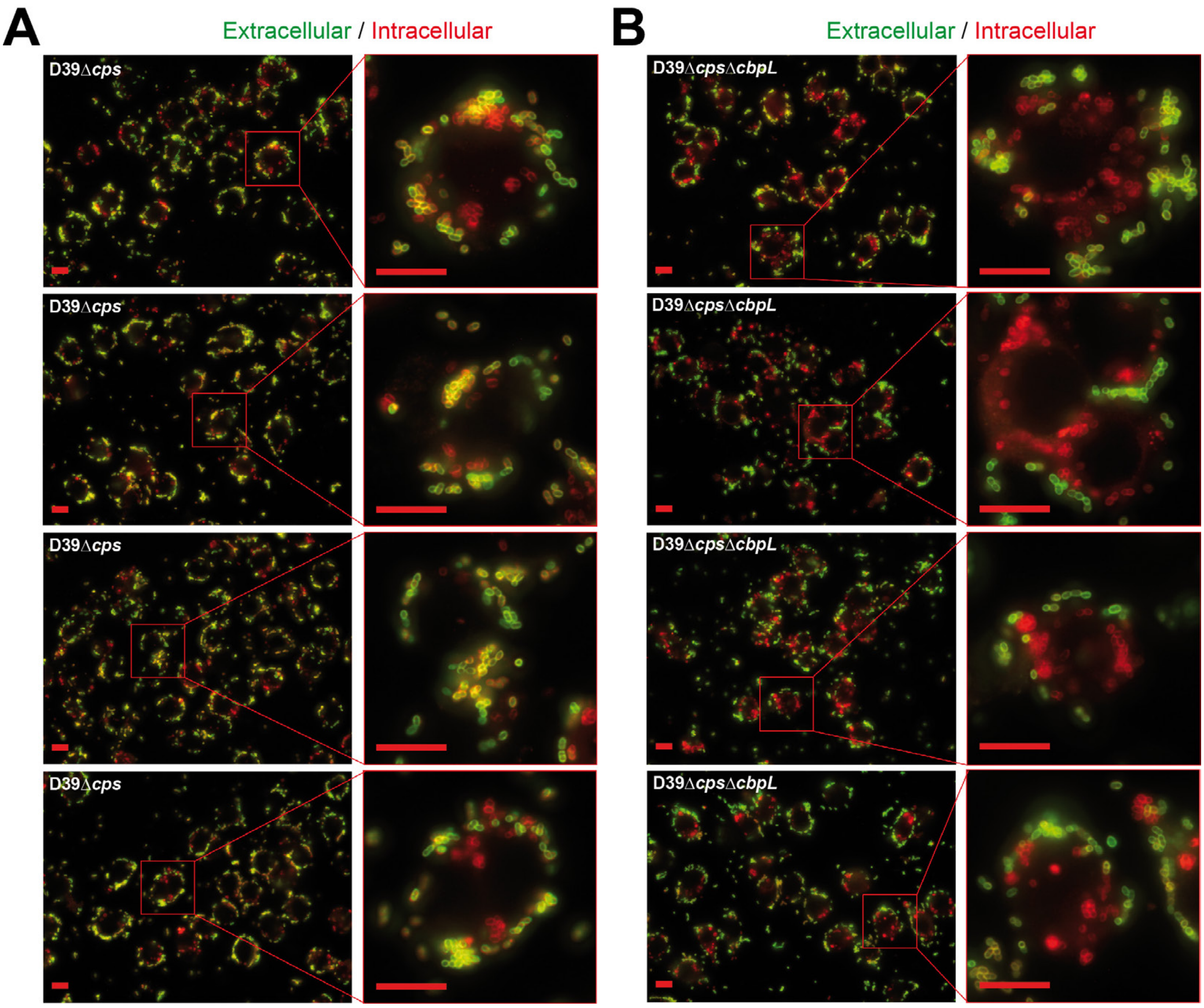

Fig. S12. Immunofluorescence microscopy of macrophage-associated and phagocytosed pneumococci.

Representative immunofluorescence microscopic images of D39Δ*cps* (**A**) and CbpL-deficient D39Δ*cps*Δ*cbpL* (**B**) pneumococci attached (green) to J774 macrophages and internalized by J774 macrophages (red) 30 min post infection with an MOI of 50 bacteria per macrophage. Extracellular pneumococci were stained with goat anti-rabbit Alexa Fluor 488 (green) after using anti-pneumococcal polyclonal antibodies as the first antibody, while intracellular pneumococci were stained after Triton™ X-100 permeabilization with goat anti-rabbit Alexa Fluor 568 (red) post Triton X-100

treatment using again rabbit anti-pneumococcal polyclonal antibodies as the first antibody. Bar represents 10µm.

```
CbpL_ltp   EYSAALGKAKSYNSLFHMSKKRMYRQLTSDFDKFSNDAAQYAIDHLDD
           ::  :. ::: : :  ::::. .  :: : :::.: ::. ::...  :
4eqq_ltp   EYRTAVSKAKQYASTVHMSKEELRSQLVS-FDKYSQDASDYAVENSGD
```

50% identity
66.7% similarity

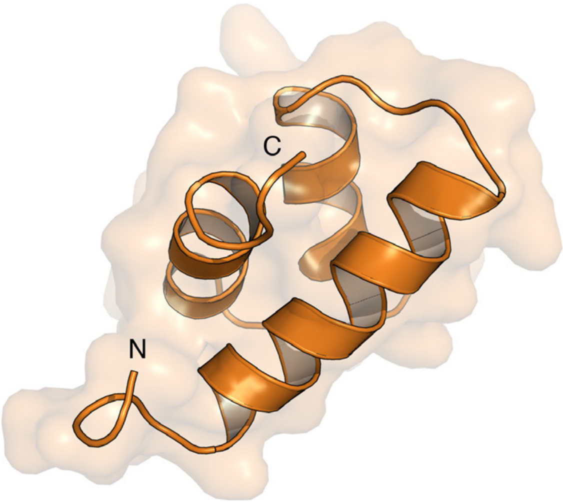

Fig. S13. Homology modeling of CbpL Ltp_Lipoprotein domain.

Homology modeling was performed using the online Swiss-Prot server using $Ltp_{TP\text{-}J34}$ protein (PDB code 4EQQ) from temperate phage TP-J34 of *Streptococcus thermophilus* as template [3-5]. Sequence alignment was performed using ClustalW.

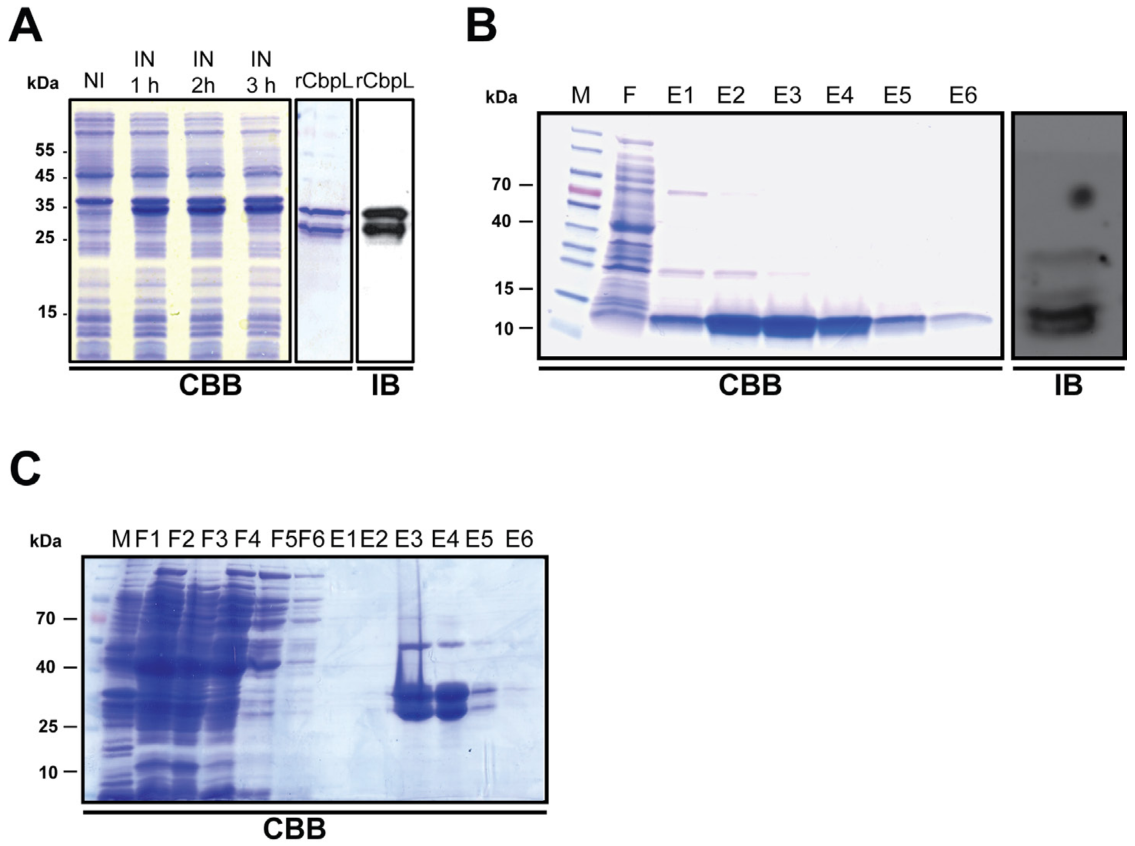

Fig. S14. Heterologous expression und purification of His_6-tagged CbpL, His_6-tagged Excalibur domain, and His_6-tagged Excalibur-CBM domain of CbpL.

(A) Heterologous expression of CbpL lacking the signal peptide (aa 27-332), (**B**) Excalibur domain (aa 27-70), and (**C**) Excalibur-CBM (aa 27- 270) cloned into expression vector pTP1 and induced with 1 mM IPTG. The His_6-tag of the purified proteins was removed by cleavage with the TEV protease. The proteins were stained with Comassie brilliant blue (CBB) and the immunoblots (IB) were performed with anti-CbpL polyclonal antibodies generated against rCbpL. NI, non-induced; IN, induced; rCbpL, recombinant without His_6-tag. F: flow through and E: protein elution fractions.

References

1 Chimalapati, S. *et al.* Infection with Conditionally Virulent Streptococcus pneumoniae Delta pab Strains Induces Antibody to Conserved Protein Antigens but Does Not Protect against Systemic Infection with Heterologous Strains. *Infect Immun* **79**, 4965-4976, doi:10.1128/Iai.05923-11 (2011).

2 Schulz, C. *et al.* Regulation of the arginine deiminase system by ArgR2 interferes with arginine metabolism and fitness of Streptococcus pneumoniae. *MBio* **5**, doi:10.1128/mBio.01858-14 (2014).

3 Arnold, K., Bordoli, L., Kopp, J. & Schwede, T. The SWISS-MODEL workspace: a web-based environment for protein structure homology modelling. *Bioinformatics* **22**, 195-201, doi:10.1093/bioinformatics/bti770 (2006).

4 Guex, N. & Peitsch, M. C. SWISS-MODEL and the Swiss-PdbViewer: an environment for comparative protein modeling. *Electrophoresis* **18**, 2714-2723, doi:10.1002/elps.1150181505 (1997).

5 Guex, N., Peitsch, M. C. & Schwede, T. Automated comparative protein structure modeling with SWISS-MODEL and Swiss-PdbViewer: a historical perspective. *Electrophoresis* **30 Suppl 1**, S162-173, doi:10.1002/elps.200900140 (2009).

6 Saleh, M. *et al.* Molecular architecture of Streptococcus pneumoniae surface thioredoxin-fold lipoproteins crucial for extracellular oxidative stress resistance and maintenance of virulence. *EMBO Mol Med* **5**, 1852-1870, doi:10.1002/emmm.201202435 (2013).

7 Holmes, A. R. *et al.* The pavA gene of Streptococcus pneumoniae encodes a fibronectin-binding protein that is essential for virulence. *Mol Microbiol* **41**, 1395-1408 (2001).

8 Tettelin, H. *et al.* Complete genome sequence of a virulent isolate of Streptococcus pneumoniae. *Science* **293**, 498-506, doi:10.1126/science.1061217 (2001).

9 Oggioni, M. R. *et al.* Pneumococcal zinc metalloproteinase ZmpC cleaves human matrix metalloproteinase 9 and is a virulence factor in experimental pneumonia. *Mol Microbiol* **49**, 795-805 (2003).

10 Tomasz, A. & Hotchkiss, R. D. Regulation of the Transformability of Pheumococcal Cultures by Macromolecular Cell Products. *Proc Natl Acad Sci U S A* **51**, 480-487 (1964).

11 Bagnoli, F. *et al.* A second pilus type in Streptococcus pneumoniae is prevalent in emerging serotypes and mediates adhesion to host cells. *J Bacteriol* **190**, 5480-5492, doi:10.1128/JB.00384-08 (2008).

12 Rennemeier, C. *et al.* Thrombospondin-1 promotes cellular adherence of gram-positive pathogens via recognition of peptidoglycan. *FASEB J* **21**, 3118-3132, doi:10.1096/fj.06-7992com (2007).

13 Jensch, I. *et al.* PavB is a surface-exposed adhesin of Streptococcus pneumoniae contributing to nasopharyngeal colonization and airways infections. *Mol Microbiol* **77**, 22-43, doi:10.1111/j.1365-2958.2010.07189.x (2010).

14 Pribyl, T. *et al.* Influence of impaired lipoprotein biogenesis on surface and exoproteome of Streptococcus pneumoniae. *J Proteome Res* **13**, 650-667, doi:10.1021/pr400768v (2014).

15 Schulz, C. *et al.* Regulation of the arginine deiminase system by ArgR2 interferes with arginine metabolism and fitness of Streptococcus pneumoniae. *MBio* **5**, doi:10.1128/mBio.01858-14 (2014).

16 Hammerschmidt, S., Tillig, M. P., Wolff, S., Vaerman, J. P. & Chhatwal, G. S. Species-specific binding of human secretory component to SpsA protein of Streptococcus pneumoniae via a hexapeptide motif. *Mol Microbiol* **36**, 726-736 (2000).

Table S1. Strain and plasmid list

Strain or plasmid	Serotype and relevant Genotype[a]	Resistance	Source or Reference
Streptococcus pneumoniae			
SP37	35A	None	NCTC10319
SP39	3	None	ATCC 6303
SP70	12F	None	MUD[b] [6]
SP129	19F	None	MUD[b] [6]
SP173 (R800)	NC[c]	None	[7]
SP257 (D39)	2	None	NCTC7466
SP261 (TIGR4)	4	None	[8]
SP309 (G54)	19F	None	[9]
SP313 (R6)	NC[c]	None	[10]
SP332	1	None	[11]
PN111	D39*Δcps*	Km^r	[12]
PN149	D39*lux*	Km^r	[13]
PN220	D39*ΔcpsΔlgt*	Km^r, Erm^r	[14]
PN319	D39Δ*cps*Δ*cbpL*	Km^r, Erm^r	This work
PN329	D39*lux*Δ*cbpL*	Km^r, Erm^r	This work
PN259	TIGR4*Δcps*	Km^r, Erm^r	[15]
PN443	TIGR4*ΔcpsΔlgt*	Km^r, Erm^r	This work
PN249	TIGR4Δ*cps*Δ*cbpL*	Km^r, Erm^r	This work
Escherichia coli			
DH5α	*Δ(lac)U169, endA1, gyrA46, hsdR17, Φ80Δ(lacZ)M15, recA1, relA1, supE44, thi-1*	None	Novagen
BL21 (DE3)	*E.coli B, F- dcm ompT hsdS gal λ (DE3), T7 polymerase gene under control of the lacUV5 promoter*	None	Stratagene
**Plasmids**			
pGEM-T Easy	TA cloning vector for PCR products; Ap^r	Ap^r	Promega
pE89	pCR2.1Topo derivative with erythromycin cassette	Ap^r, Km^r , Erm^r	[16]
p562	pGEM-T derivative with *sp_0667* + 5´and 3´ flanking region for mutagenesis (TIGR4-derived strains)	Ap^r	This work
p569	pGEM-T derivative with *sp_0667* interrupted by Erm resistance cassette (TIGR4-derived strains)	Ap^r, Erm^r	This work
p716	pGEM-T derivative with *spd_0579* + 5´and 3´ flanking region for mutagenesis (D39-derived strains)	Ap^r	This work
p717	pGEM-T derivative with *spd_0579* interrupted by Erm resistance cassette (D39-derived strains)	Ap^r, Erm^r	This work
pET28a	Protein expression vector	Km^r	Novagen
pTP1	pET28a derivative expression vector	Km^r, Erm^r	[6]
p630	pET28TEV derivative with TIGR4 *sp_0667* (*cbpL*) for protein production and mice immunization	Km^r	This work
p718	pET28TEV derivative with TIGR4 *sp_0667* (*cbpL*) for protein production	Km^r	This work
p1025	pET28TEV derivative with TIGR4 *sp_0667* w/o Ltp for protein production	Km^r	This work
p1070	pET28TEV derivative with TIGR4 Excalibur_*sp_0667* for protein production	Km^r	This work

[a]Ap, ampicillin; Km, kanamycin; Erm, erythromycin; r, resistant

[b]MUD, Medical University of Düsseldorf, Germany

[c]NC, nonencapsulated strain

Table S2. Primer list

Primer intended use	Primer name	Sequence (5'-3')
Insertion-deletion mutagenesis		
Amplification of *sp_0667* + 5´and		
3´ flanking region (D39-derived strains)	CbpL_632	5`-ACTAGAATTCGACAAAAGATAGAGGCGGA-3`
	CbpL_409	5`-AACCTTCCAAGCTGCAGCTGCTGCACCAGCAACA-3`
Amplification of *sp_0667* + 5´and		
3´ flanking region (TIGR4-derived strains)	CbpL_406	5`-CTACTACTAGAATTCTGCATGGGTTAGGGCAGT-3`
	CbpL_409	5`-AACCTTCCAAGCTGCAGCTGCTGCACCAGCAACA-3`
Inverse PCR of *sp_0667* + 5´and		
3´ flanking region (pGEMTeasy)	CbpL _408	5`-ACTCACTCACTGCTCGAGTGCAGCTCAATATGCCATT-3`
	CbpL _407	5`-ATCATCATCATCGGGTACCCGTTTATTCATTTCTTTCTCCCATA-3`
Antibiotic cassette amplification		
erythromycin (*ermB*)	ermB_105	5`-GATGATGATGATCCCGGGTACCAAGCTTGAATTCACG GTTCGTGTTCGTGCTG-3`
	ermB_106	5`-AGTGAGTGAGTCCCGGGCTCGAGAAGCTTGA ATTCGTAGGCGCTAGGGACCTC-3`
Recombinant protein production and vector modification		
sp_0667 (TIGR4) His-tag-protein	CbpL _461	5`-GCGCGCTAGCGAAGAAAACATCCATTTTTC-3`
rCbpL	CbpL _462	5`-GGCCGAGCTCTTAATCATCTAAATGATCAATGG-3`
sp_0667 (TIGR4) untagged protein	CbpL _598	5`-GCGCCCATGGAAGAAAACATCCATTTTTC-3`
rCbpL	CbpL _462	5`-GGCCGAGCTCTTAATCATCTAAATGATCAATGG-3`
sp_0667 (TIGR4)	CbpL _461	5`-GCGCCCATGGAAGAAAACATCCATTTTTC-3`
Excalibur domain His-tag-protein	CbpL _1156	5`- GGCCGAGCTCTAATTTTTCAATTCGCAAGCCAC -3`
sp_0667 (TIGR4)	CbpL _461	5`-GCGCCCATGGAAGAAAACATCCATTTTTC-3`
Excalibur-CBM (w/o Ltp) His-tag-protein	CbpL _1321	5`- GCGCGCGAGCTCGCTATCATTACTAGAAGAAGCT -3`
sp_0667 (TIGR4)	CbpL _1470	5`-GCGCGCGCTAGCGCTCCTAAGGGTGCTTTTAA-3`

[a] Restriction sites are underlined
